# Generalized Exponential Distribution in Flood Frequency Analysis for Polish Rivers

**DOI:** 10.1371/journal.pone.0143965

**Published:** 2015-12-10

**Authors:** Iwona Markiewicz, Witold G. Strupczewski, Ewa Bogdanowicz, Krzysztof Kochanek

**Affiliations:** 1 Department of Hydrology and Hydrodynamics, Institute of Geophysics Polish Academy of Sciences, Warsaw, Poland; 2 CHIHE Norway Grants, Institute of Geophysics Polish Academy of Sciences, Warsaw, Poland; Brown University, UNITED STATES

## Abstract

Many distributions have been used in flood frequency analysis (FFA) for fitting the flood extremes data. However, as shown in the paper, the scatter of Polish data plotted on the moment ratio diagram shows that there is still room for a new model. In the paper, we study the usefulness of the generalized exponential (GE) distribution in flood frequency analysis for Polish Rivers. We investigate the fit of GE distribution to the Polish data of the maximum flows in comparison with the inverse Gaussian (IG) distribution, which in our previous studies showed the best fitting among several models commonly used in FFA. Since the use of a discrimination procedure without the knowledge of its performance for the considered probability density functions may lead to erroneous conclusions, we compare the probability of correct selection for the GE and IG distributions along with the analysis of the asymptotic model error in respect to the upper quantile values. As an application, both GE and IG distributions are alternatively assumed for describing the annual peak flows for several gauging stations of Polish Rivers. To find the best fitting model, four discrimination procedures are used. In turn, they are based on the maximized logarithm of the likelihood function (*K* procedure), on the density function of the scale transformation maximal invariant (*QK* procedure), on the Kolmogorov-Smirnov statistics (*KS* procedure) and the fourth procedure based on the differences between the ML estimate of 1% quantile and its value assessed by the method of moments and linear moments, in sequence (*R* procedure). Due to the uncertainty of choosing the best model, the method of aggregation is applied to estimate of the maximum flow quantiles.

## Introduction

Flood frequency analysis (FFA) provides information about the probable size of flood flows and has been used for the design of civil engineering works over the century. The assessment of the flood (upper) quantiles is required for dimensioning hydraulic structures affected by high waters, such as culverts, dams, bridges, overflow channels, spillways, levees, and others. FFA plays an important role in reducing the flood risk, since the flood quantile estimates are essential in determining the limits of flood zones with varying degree of flood risk as well in estimating the risk of exploitation of floodplains.

The quantile of the order of *F* ∈ (0,1) is defined as the value *x*_*F*_ satisfying the equation:

$$\int_{-\infty }^{x_{F}}f\left(x\right)dx=F$$ (1)

where *f* is the probability density function (PDF) of the continuous random variable. The flood (upper) quantile means the probable maximum flow of the return period of *T* years and the relation between the probability of non-exceedance *F* and return period *T* has the form:

$$T=\frac{1}{1-F}$$ (2)

Since the return period *T* equal to 100, 500, 1000 is usually used, then the probability *F* is close to 1, i.e. close to its highest value. Equivalently, the probabilities of exceedance *p* can be applied, where:

p=1−F (3)

The at-site frequency analysis is the most commonly used approach. Then the estimation of flood quantiles refers to the choice of the form of probability density function describing the annual peak flows for the investigated gauging station. The distribution function assumed (also called the model) has a character of statistical hypothesis. Simultaneously, the method of estimation of parameters and, thus, upper quantiles of the assumed distribution is selected. This step is denoted D/E for “distribution and estimation procedure”. To find the best fitting model to the empirical data, the chosen discrimination procedure is applied.

The choice of distribution for fitting the annual maximum flows has attracted considerable interest, e.g. [1–7] and many others. According to the hydrological report of the World Meteorological Organization from 1989 [8], the Gumbel and log-normal distributions were the most commonly used for the description of the peak flow data. In Poland, the Pearson III type distribution has been recommended by Central Office of Water Management for national use [9]. These regulations are still in force, although other models are also applied in practice. Nowadays in many countries around the world, the heavy-tailed distributions are recommend for modelling of extreme flow series, e.g. [10–15]. The heavy-tailed distributions have conventional moments only in a certain range, which decreases with growing moment order. However, the heavy-tailed form of hydrological variables is not sufficiently supported, e.g. [16], [17]. Moreover, the analysis of Polish datasets of annual peak flows in [18] shows that they should be modeled using soft-tailed rather than heavy-tailed distributions.

The characteristics describing properties of the distribution are the summary statistics. Several systems of summary statistics have been developed. Based on different principles they provide, in particular, the measures of location, dispersion, skewness and kurtosis. The summary statistics calculated for a random sample consecutively serve for identifying and fitting PDFs. Among the systems of summary statistics, the most popular are the system of conventional moments (*μ*_*r*_) and that of linear moments, called *L*-moments (*λ*_*r*_), presented in Table 1 along with the dimensionless versions of the summary statistic sets in the form of summary statistic ratios (in square brackets). It is convenient to use the dimensionless versions of the summary statistics, since they measure the shape of a distribution independently of its scale of measurement.

**Table 1:** Summary statistics according to the system of conventional and linear moments.

System of summary statistics	Location measure	Dispersion measure [Dimensionless]	Skewness measure [Dimensionless]
Conventional moments	$\mu =\int_{-\infty }^{+\infty }xdF\left(x\right)$	$\mu_{2}=\int_{-\infty }^{+\infty }{\left(x-\mu \right)}^{2}dF\left(x\right)$ $\left[C_{V}=\mu_{2}^{1/2}/\mu \right]$	$\mu_{3}=\int_{-\infty }^{+\infty }{\left(x-\mu \right)}^{3}dF\left(x\right)$ $\left[C_{S}=\mu_{3}/\mu_{2}^{3/2}\right]$
Linear moments	*λ*_1_ = *β*_0_ ≡ *μ* $\beta_{r}=\int_{-\infty }^{+\infty }xF^{r}\left(x\right)\;dF\left(x\right)$	$\lambda_{2}=2\beta_{1}-\beta_{0}=\int_{-\infty }^{+\infty }2\left(x-\mu \right)F\left(x\right)\;dF\left(x\right)$ [*LC*_*V*_ = *λ*_2_ / *λ*_1_]	*λ*_3_ = 6*β*_2_ − 6*β*_1_ + *β*_0_ [*LC*_*S*_ = *λ*_3_ / *λ*_2_]

As seen from Table 1, the *L*-moments can be defined by the probability weighted moments of a random variable *β*_*r*_ for *r* = 0,1,2,… [19]. The *L*-moments create an attractive system because their estimators, in contrast to the classical moments estimators, are not biased and the sampling *L*-moment ratios have very small biases for moderate and large samples.

For the convenience of the reader, the abbreviations and symbols commonly used in the paper are gathered in Table in S1 Table.

**Fig 1:**
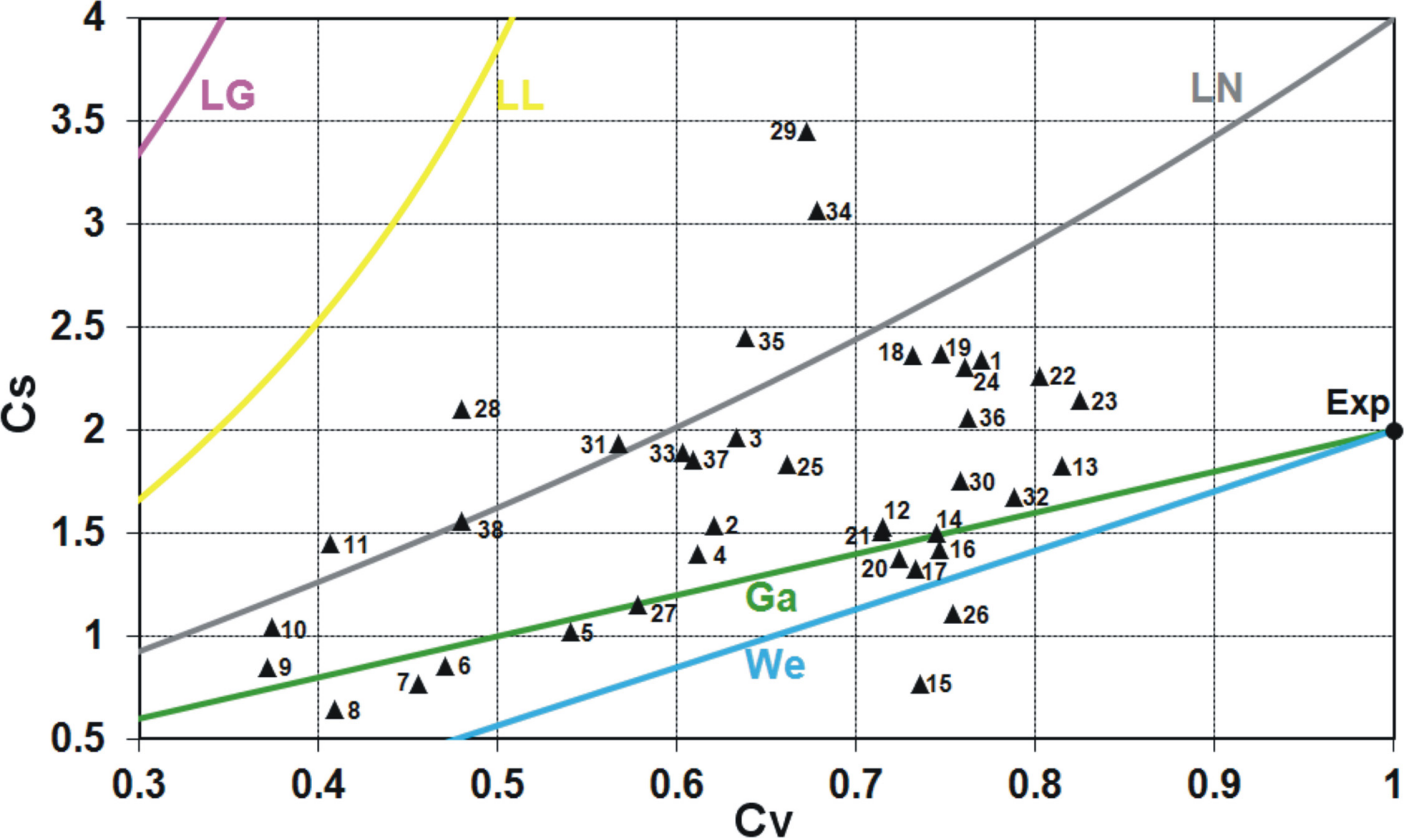
The relation of conventional skewness coefficient *C*_*S*_ versus conventional variation coefficient *C*_*V*_ for some two-parameter distributions commonly used if FFA plotted with the Polish data of 90-year annual peak flow series. Distributions: Ga–gamma, We–Weibull, LN–log-normal, LL–log-logistic, LG–log-Gumbel, Exp–exponential.

**Fig 2:**
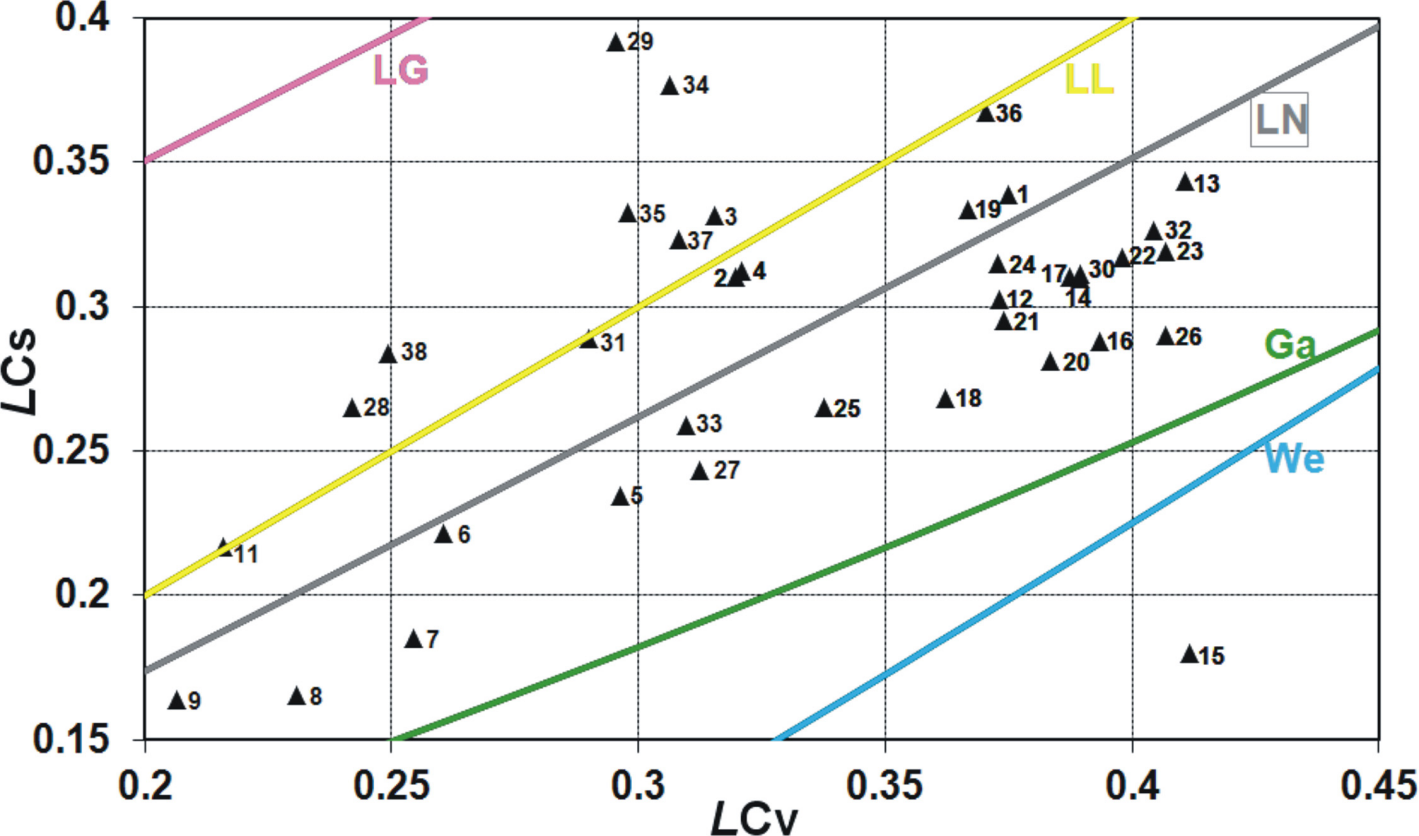
The relation of linear skewness coefficient *LC*_*S*_ versus linear variation coefficient *LC*_*V*_ for some two-parameter distributions commonly used if FFA plotted with the Polish data of 90-year annual peak flow series. Distributions: Ga–gamma, We–Weibull, LN–log-normal, LL–log-logistic, LG–log-Gumbel, Exp–exponential.

For two-parameter distributions lower bounded at zero, a basic illustration that provides an intuition to a practitioner to distinguish various distributions is the graph of the relationship between the conventional variation coefficient *C*_*V*_ and the conventional skewness coefficient *C*_*S*_ or between their linear counterparts, i.e. between the linear variation coefficient *LC*_*V*_ and the linear skewness coefficient *LC*_*S*_. These relationships show in what range of *C*_*V*_ − *C*_*S*_ various distributions can be used, e.g. the log-logistic and log-Gumbel distributions are not proper for modelling the data series of small skewness *C*_*S*_ < 1 and average variation *C*_*V*_ > 0.5**.** Both relations, *C*_*V*_ − *C*_*S*_ and *LC*_*V*_ − *LC*_*S*_, are shown in Figs 1 and 2, respectively, for two-parameter distributions commonly used in FFA (lines) plotted with the Polish data of annual peak flows for 38 gauging stations (triangular points). To find the data availability, see the Acknowledgment.

In Figs 1 and 2, if some point lies on the line corresponding to certain distribution or around it, it may indicate that this distribution will be the best fitting to the data series. However, the perfect fit would hold for the asymptotic sample from a given distribution. Due to a limited length of the data series, such graphical analysis is only preliminary and the distribution best fitted to the data is indicated by the discrimination procedures, which will be discussed later in this paper.

**Fig 3:**
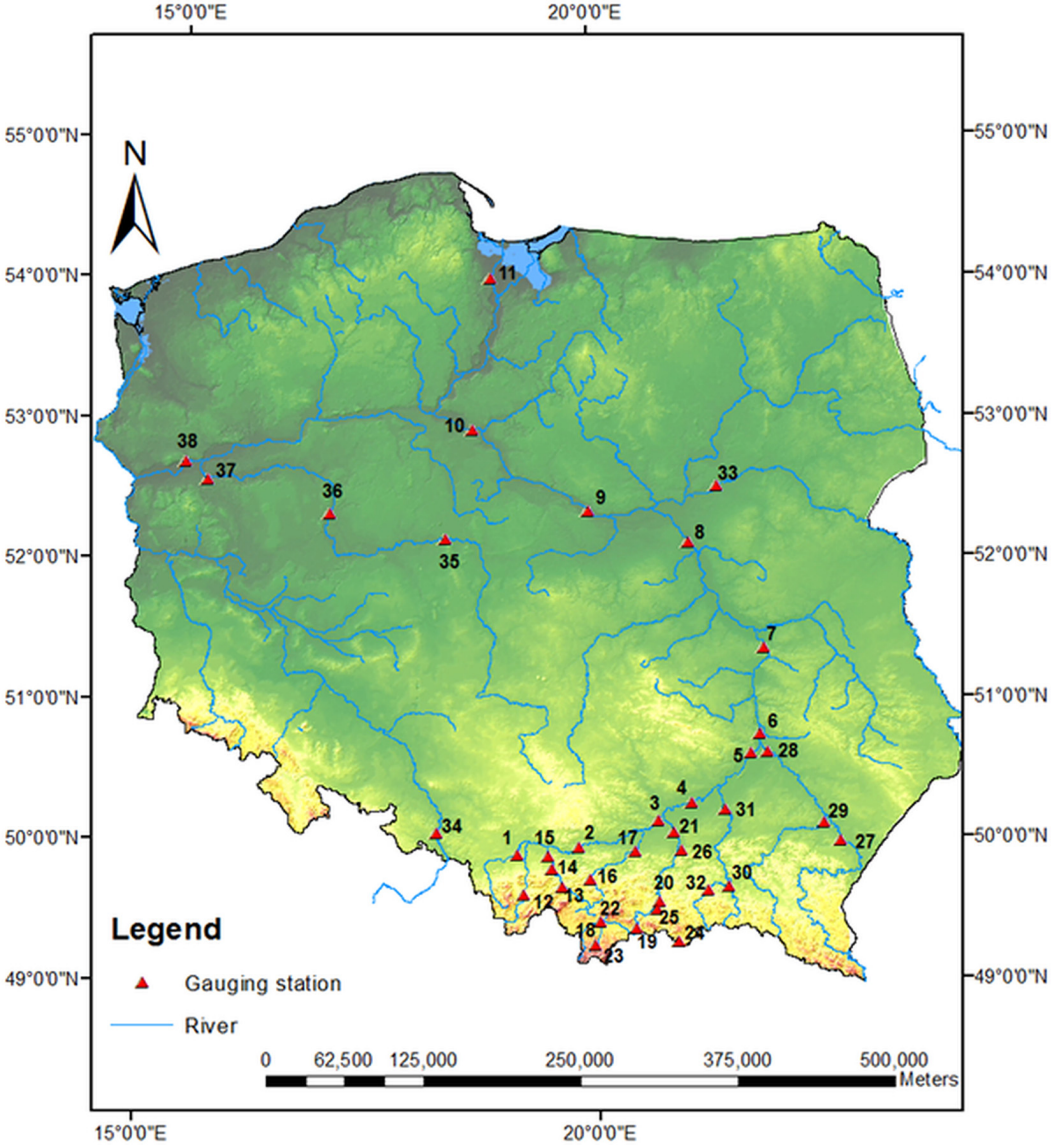
Map of 38 Polish gauging stations.

The respective measuring sections are listed in Table 2 and illustrated in Fig 3. Most of analyzed data series cover the period 1921–2010.

**Table 2:** Origin and basic characteristics of 38 Polish gauging stations.

Basin/River	Gauging station		Drainage area (10^3^ km^2^)	Average peak flow (m^3^/s)	Variation coefficient (*Cv*)	Skewness coefficient (*C*_*S*_)
	No. Name					
Vistula	1	Jawiszowice	0.971	157.6	0.770	2.3388
	2	Tyniec	7.520	709.0	0.620	1.5344
	3	Jagodniki	12.06	1159.	0.633	1.9630
	4	Szczucin	23.90	1941.	0.612	1.3989
	5	Sandomierz	31.85	2334.	0.541	1.0175
	6	Zawichost	50.73	3328.	0.471	0.8530
	7	Puławy	57.26	3064.	0.455	0.7697
	8	Warsaw	84.54	3023.	0.409	0.6450
	9	Kępa	169.0	3803.	0.371	0.8461
	10	Toruń	181.0	3817.	0.374	1.0407
	11	Tczew	194.4	3635.	0.407	1.4483
Vistula/Sola	12	Żywiec	0.785	322.8	0.715	1.5249
Vistula/Skawa	13	Sucha	0.468	171.0	0.814	1.8293
	14	Wadowice	0.835	271.3	0.7446	1.4980
Vistula/Skawa/Wieprzówka	15	Rudze	0.154	57.28	0.7359	0.7649
Vistula/Raba	16	Stróża	0.644	222.9	0.7463	1.4184
	17	Proszówki	1.470	451.0	0.7330	1.3251
Vistula/Dunajec	18	Kowaniec	0.681	254.7	0.7311	2.3639
	19	Krościenko	1.580	448.4	0.7474	2.3732
	20	Nowy Sącz	4.340	956.6	0.7239	1.3761
	21	Żabno	6.740	1165.	0.7138	1.5067
Vistula/Dunajec/ Czarny Dunajec	22	Nowy Targ	0.432	166.3	0.802	2.2641
Vistula/Dunajec/Biały Dunajec	23	Zakopane	0.058	39.09	0.8245	2.1484
Vistula/Dunajec/Poprad	24	Muszyna	1.510	234.8	0.7602	2.3053
	25	Stary Sącz	2.070	323.2	0.6611	1.8310
Vistula/Dunajec/Biała	26	Koszyce Wlk.	0.957	280.6	0.7543	1.1083
Vistula/San	27	Jarosław	7.040	749.6	0.5782	1.1508
	28	Radomyśl	16.80	956.5	0.4800	2.0993
Vistula/San/Wisłok	29	Tryńcza	3.520	246.3	0.6723	3.4536
Vistula/Wisłoka	30	Żółków	0.581	175.6	0.7582	1.7529
	31	Mielec	3.690	546.2	0.5669	1.9336
Vistula/Wisłoka/Ropa	32	Klęczany	0.482	125.8	0.7882	1.6732
Vistula/Bug	33	Wyszków	39.10	601.2	0.6030	1.8896
Oder	34	Miedonia	6.740	616.7	0.6784	3.0644
Oder/Warta	35	Konin	13.40	252.8	0.6384	2.4472
	36	Poznań	25.90	420.5	0.7626	2.0603
	37	Skwierzyna	32.10	384.6	0.6090	1.8565
	38	Gorzów	52.40	512.8	0.4793	1.5588

**Fig 4:**
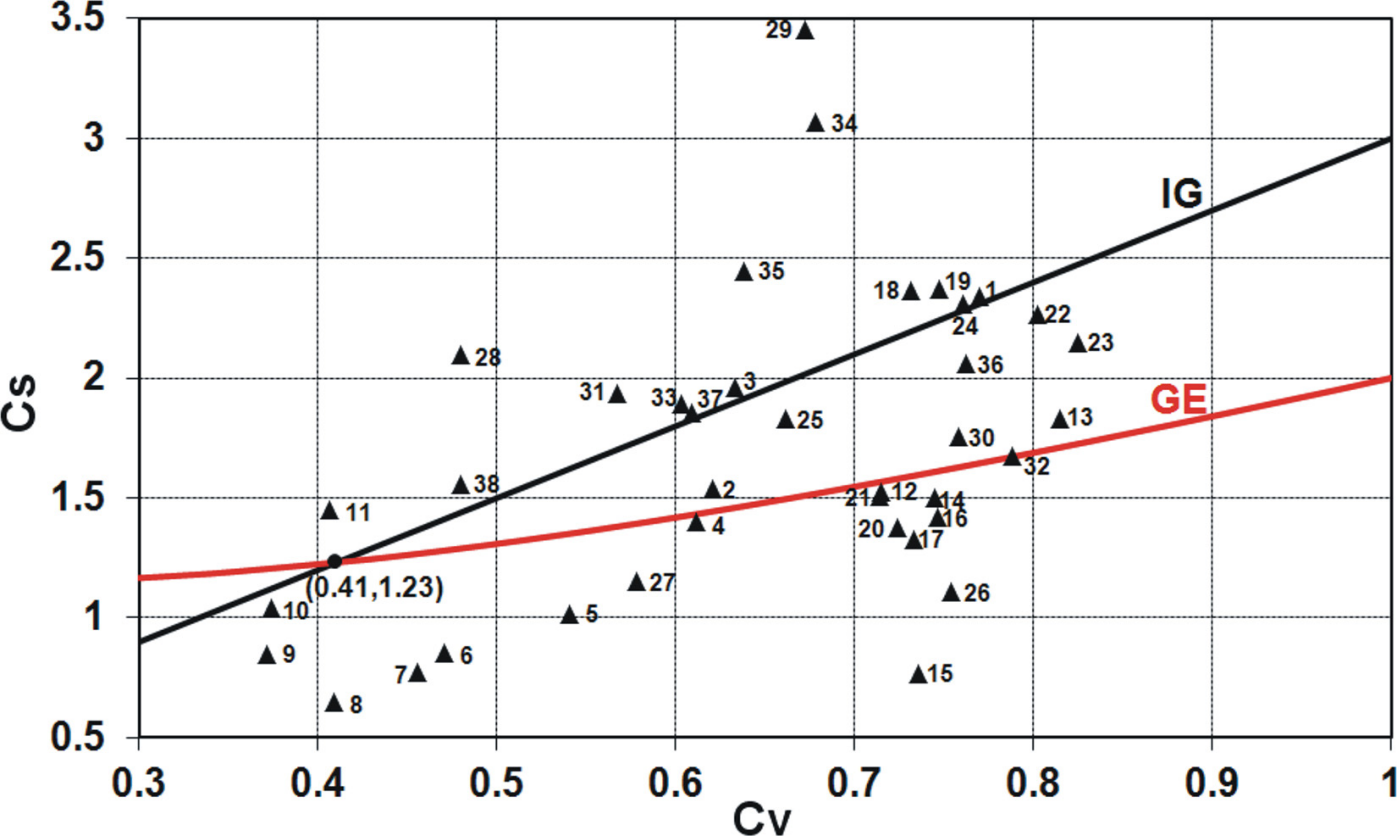
The relation of conventional skewness coefficient *C*_*S*_ versus conventional variation coefficient *C*_*V*_ for two-parameter inverse Gaussian, IG, and generalized exponential, GE, distributions plotted with the Polish data of 90-year annual peak flow series.

**Fig 5:**
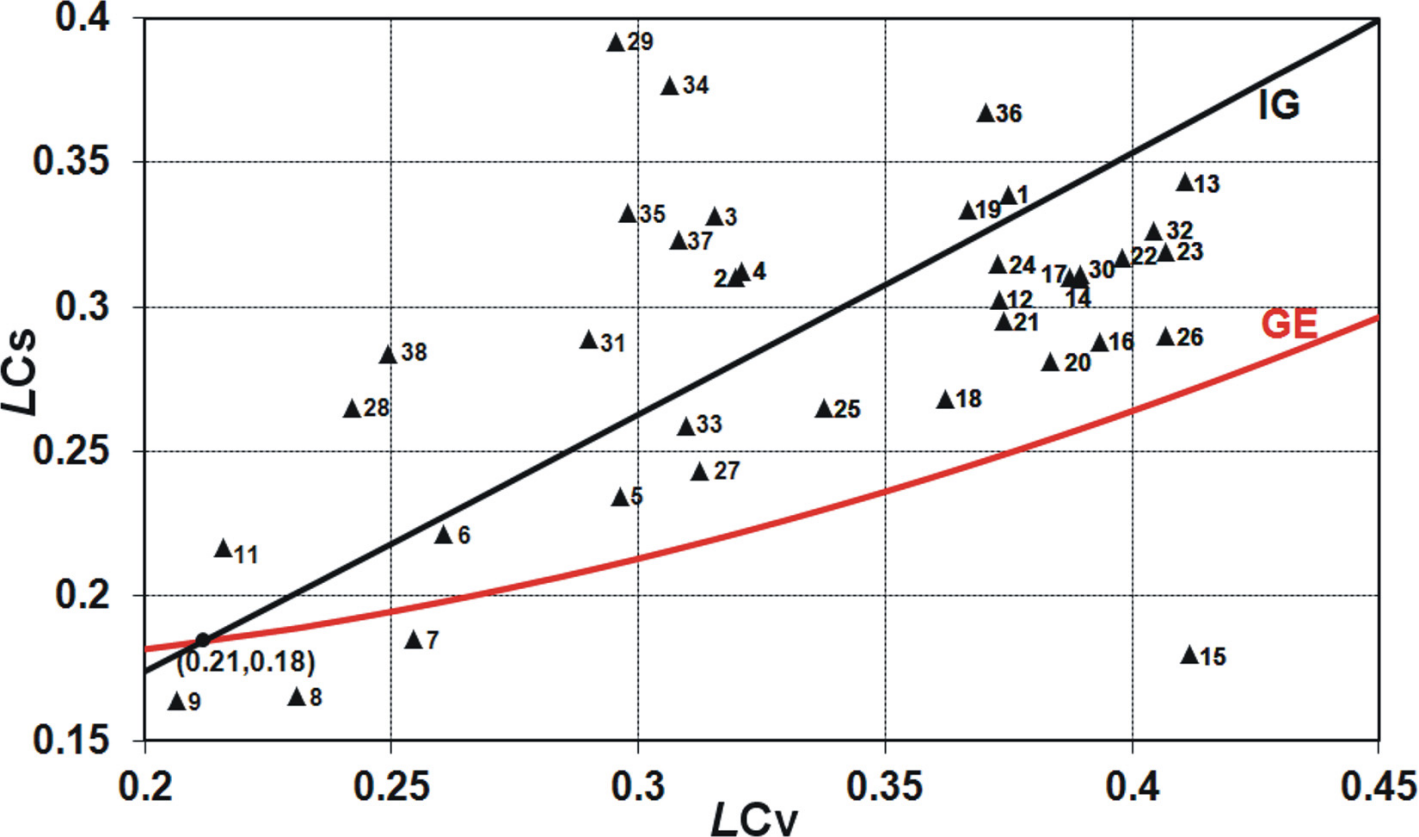
The relation of linear skewness coefficient *LC*_*S*_ versus linear variation coefficient *LC*_*V*_ for two-parameter inverse Gaussian, IG, and generalized exponential, GE, distributions plotted with the Polish data of 90-year annual peak flow series.

As seen in Figs 1 and 2, for both conventional and linear moments ratios, there is a range of values taken by the Polish data series and not covered by any distribution. Clearly, there is still room for a new model. The inverse Gaussian (IG) and the generalized exponential (GE) distributions with the scale and shape parameters seem to be a suitable complement (see Figs 4 and 5), i.e. there are many points *C*_*V*_-*C*_*S*_ corresponding to the Polish data series, which are on or around the lines of IG and GE distributions.

The GE distribution is used quite effectively to analyze lifetime data in the reliability analysis, being an alternative to the two-parameter gamma, Weibull, Pareto and log-normal distributions [20]. The aim of the study is to assess the usefulness of the generalized exponential distribution in flood frequency analysis for Polish Rivers, as a complementary to the inverse Gaussian distribution, which has proved to be suitable for many Polish data series [21], [22], [18]. In the paper, two-parameter distributions, instead of their three-parameter counterparts, are used for the modelling of relatively large-size samples (i.e. 90 elements), since our studies are intended to be applicable for most of the available observation series, which are much shorter than those investigated here. The short length of the data series hinders the proper selection of the distribution and two-parameter PDFs are usually used for their modelling. To reduce the uncertainty in the estimation of the extreme value distribution quantiles, the multi-model approach proposed by Bogdanowicz [23] is applied.

The paper is organized as follows. After providing some introduction to the topic, the probability distributions analyzed in the paper are shortly presented in second section. Next, the four discrimination procedures used to select the best fitting model are shown. Sequent two sections provide the results of the simulation studies on the probability of correct selection (PCS) among the GE and IG distributions along with the analysis of the asymptotic model error in respect to the upper quantile. In the case study section, fitting the GE and IG distributions to the 90-year series of annual maximum flows is compared for four selected gauging stations of Polish Rivers. Then, the method of aggregated quantiles is proposed for evaluation of upper quantile values. The paper is concluded in the final section.

## GE and IG Probability Distributions

The inverse Gaussian (known also under the name of Wald) distribution has several properties analogous to the Gaussian distribution. In fact, the name is misleading, since it is “inverse” only in that, while the Gaussian describes the distribution of distance at a fixed time in Brownian motion, the inverse Gaussian describes PDF of the first passage time for a Brownian motion starting at zero to reach the absorbing barrier at the fixed point [24]. The same function appears in linear flood routing modelling as the impulse response of the semi-infinite channel at a fixed distance for the Froude number equal to zero [25], [26], and the name “linear convective diffusion model” for IG has been used in FFA [27–29]. In the last paper, the similarity between IG and LN distributions was shown by comparison of their first five moment estimates. Moreover, fitting of the two distributions to Polish data was compared there by the likelihood ratio. It indicates the preference of the IG model over the LN model for 27 out of 39 annual peak flow series. The simulation studies on the probability of correct selection among IG and LN have been carried out [21], adopting several discrimination statistics. The discrimination procedures based on the likelihood ratio and the *R* statistics [22] favor IG over LN, while the discrimination procedure based on the *QK* statistics [30] favors LN over IG. Investigation of Polish annual maxima datasets by the *L*-moment ratio diagrams and the test of linearity on log–log plots shows that the inverse Gaussian distribution represents flood frequency characteristics of Polish Rivers quite well, in particular of lowland rivers [18].

The generalized exponential distribution has been developed by Gupta and Kundu [31] and used quite effectively in many situations where a positive skewed distribution is needed. The closeness of GE distribution with gamma, Weibull, and log-normal distributions has been demonstrated [32–35]. The generalized exponential distribution has been applied to analyze lifetime data in the reliability analysis [20]. However, to the best of our knowledge it has not been used in FFA so far but in Poland where the GE model has been introduced for describing random properties of seasonal maximum annual flows [36].

The basic statistical characteristics of both IG and GE distributions are presented in Table 3.

**Table 3:** Basic characteristics of two-parameter IG and GE distributions.

	Generalized exponential	Inverse Gaussian
**PDF**	*f*(*x*) = *αλ*(1 − *e* ^−*λx*^)^(*α*−1)^ *e* ^−*λx*^; *λ*,*α*,*x* > 0	$f(x)=\frac{\alpha }{\sqrt{\pi \;x^{3}}}\text{exp}\left[-{\left(\alpha -\frac{\beta }{\alpha }x\right)}^{2}/x\right]$; *α*,*β*,*x* > 0
**CDF**	*F*(*x*) = (1 − *e* ^−*λx*^)^*α*^	$F\left(x\right)=\frac{1}{2}\left[2-erfc\left(\frac{-\alpha +x\beta /\alpha }{\sqrt{x}}\right)+\text{exp}\left(4\beta \right)erfc\left(\frac{\alpha +x\beta /\alpha }{\sqrt{x}}\right)\right]$
**Quantile F**	$x_{F}=-\frac{\text{ln}\left(1-F^{1/\alpha }\right)}{\lambda }$	$x_{F}={\left(\frac{\alpha }{t_{F}\left(\beta \right)}\right)}^{2}$ ^a^
**Mean**	$\mu =\frac{1}{\lambda }\left[\psi \left(\alpha +1\right)-\psi \left(1\right)\right]$ ^b^	$\mu =\frac{\alpha^{2}}{\beta }$
**Variation coefficent**	$C_{V}=\frac{\sqrt{\mu_{2}}}{\mu }=\frac{\sqrt{\psi^{\prime }\left(1\right)-\psi^{\prime }\left(\alpha +1\right)}}{\psi \left(\alpha +1\right)-\psi \left(1\right)}$ ^b^	$C_{V}=\frac{1}{\sqrt{2\beta }}$
**Skewness coefficent**	$C_{S}=\frac{\mu_{3}}{\mu_{2}^{3/2}}=\frac{\psi^{\prime \prime }\left(\alpha +1\right)-\psi^{\prime \prime }\left(1\right)}{{\left[\psi^{\prime }\left(1\right)-\psi^{\prime }\left(\alpha +1\right)\right]}^{3/2}}$ ^b^	$C_{S}=\frac{\mu_{3}}{\mu_{2}^{3/2}}=\frac{3}{\sqrt{2\beta }}=3C_{V}$
**Kurtosis**	$C_{k}=\frac{\mu_{4}}{\mu_{2}^{2}}=\frac{\psi^{\prime \prime \prime }\left(1\right)-\psi^{\prime \prime \prime }\left(\alpha +1\right)}{{\left[\psi^{\prime }\left(1\right)-\psi^{\prime }\left(\alpha +1\right)\right]}^{2}}+3$ ^b^	$C_{k}=\frac{\mu_{4}}{\mu_{2}^{2}}=3\left(\frac{5}{2\beta }+1\right)=2\left(5C_{{}_{V}}^{2}+1\right)$
**Linear variation coefficient**	$LC_{V}=\frac{\lambda_{2}}{\lambda_{1}}=\frac{\psi \left(2\alpha +1\right)-\psi \left(\alpha +1\right)}{\psi \left(\alpha +1\right)-\psi \left(1\right)}$	$LC_{V}=\frac{\lambda_{2}}{\lambda_{1}}=\mu^{-1}\int_{-\infty }^{+\infty }2F\left(x\right)\left(x-\mu \right)\;dF\left(x\right)$
**Linear skewness coefficient**	$LC_{S}=\frac{\lambda_{3}}{\lambda_{2}}=\frac{\psi \left(\alpha +1\right)-3\cdot \psi \left(2\alpha +1\right)+2\cdot \psi \left(3\alpha +1\right)}{\psi \left(2\alpha +1\right)-\psi \left(\alpha +1\right)}$	$LC_{S}=\frac{\lambda_{3}}{\lambda_{2}}=\frac{\int_{-\infty }^{+\infty }F\left(x\right)\left(3xF\left(x\right)-3x+\mu \right)\;dF\left(x\right)}{\int_{-\infty }^{+\infty }F\left(x\right)\left(x-\mu \right)\;dF\left(x\right)}$

^a^ *t*_*F*_(*β*) is the upper limit of the integral *F* given below, where Φ(⋅) is the cumulative probability of the normal distribution N(0,1):

$F=1-\frac{2}{\sqrt{\pi }}\int_{0}^{t_{F}}\text{exp}\left[-{\left(z-\frac{\beta }{z}\right)}^{2}\right]dz=\Phi \left(\sqrt{2}\left(\frac{\beta }{t_{F}}-t_{F}\right)\right)+e^{4\beta }\left\{1-\Phi \left(\sqrt{2}\left(\frac{\beta }{t_{F}}+t_{F}\right)\right)\right\}$ ^b^ *ψ*, *ψ*′, *ψ*″ and *ψ*‴ are digamma, trigamma, tetragamma and pentagamma functions, respectively.

The polygamma functions are defined as the logarithmic derivative of the gamma function [37]:

$$\psi^{\left(n\right)}\left(z\right)=\frac{d^{n}}{dz^{n}}\psi \left(z\right)=\frac{d^{n+1}}{dz^{n+1}}\text{ln}\left[\Gamma \left(z\right)\right]\;\text{for}\;n=1,2,3,\ldots$$ (4)

For real positive arguments *z*, digamma function *ψ*(*z*) is a concave increasing function of *z* which satisfies the following relation [37], [38]:

$$\psi \left(z\right)=\text{ln}\left(z\right)-\frac{1}{2z}-\frac{1}{12z^{2}}+\frac{1}{120z^{4}}-\frac{1}{252z^{6}}+\frac{1}{240z^{8}}-\frac{1}{132z^{10}}+O\left(\frac{1}{z^{12}}\right)$$ (5)

Differentiating Eq 5 appropriate number of times, one gets the evaluations of polygamma functions that can be used for numerical calculations instead of analytical formulas.

Only the first two linear moments of GE distribution have been derived so far [20]. The formula for its third linear moment (*λ*_3_) and thus for the linear skewness coefficient (*LC*_*S*_) has been derived by the authors (see Appendix) and presented in Table 3. Since the linear moments of IG distribution have no analytical form, their integral formulas are applied for computational calculations and the trapezoidal rule is used for approximation of the definite integral [39]. The details concerning the derivation of the formula for the quantile corresponding to probabilities of non-exceedance *F* (*x*_*F*_) for IG distribution are shown in [27].

**Fig 6:**
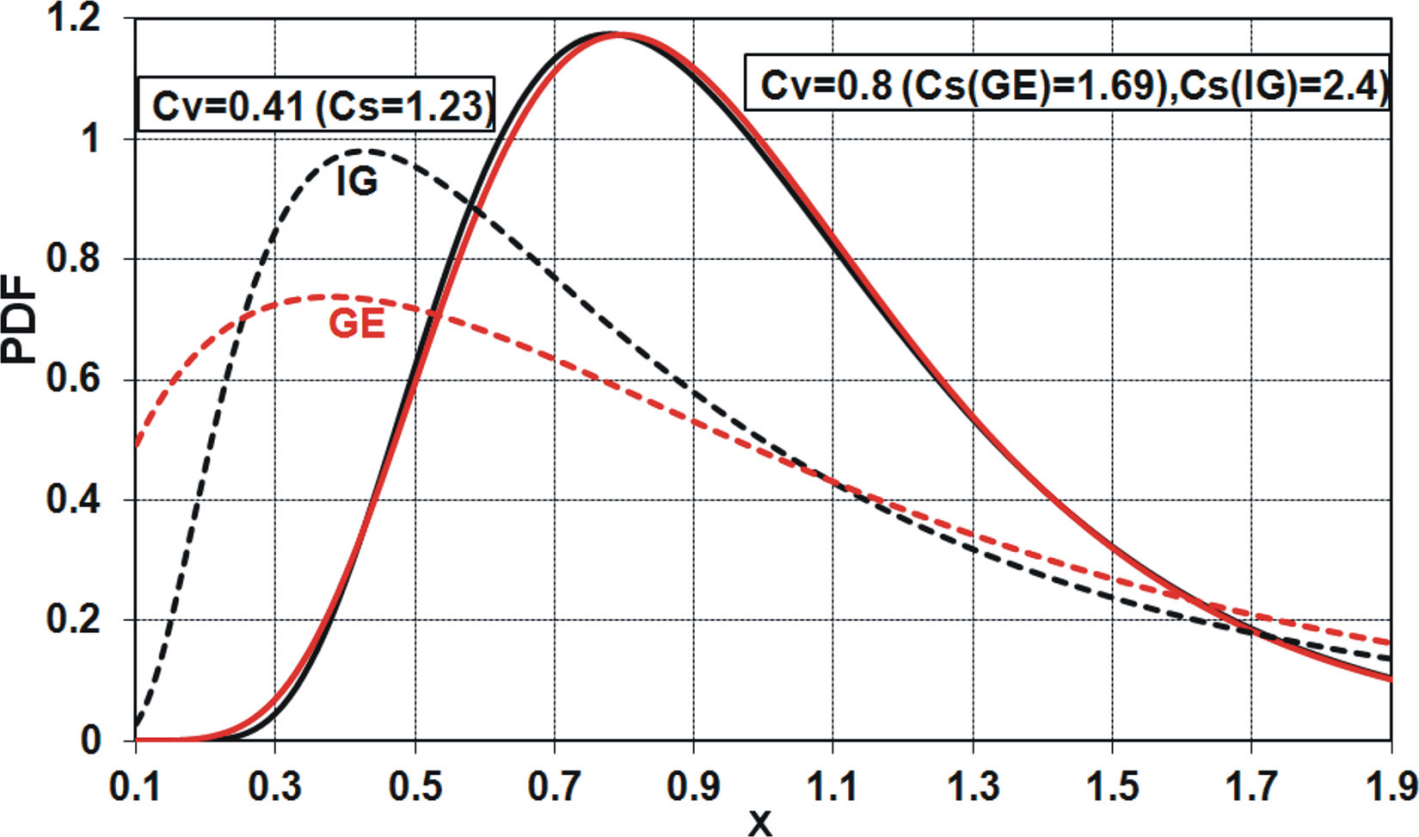
Probability density functions of GE and IG distributions for *μ* = 1.0 and selected values of *C*_*V*_ and thus *C*_*S*_.

As shown in Fig 4, for the variation coefficient *C*_*V*_ equal to 0.41, the skewness coefficients *C*_*S*_ of both GE and IG distributions are the same and amount to 1.23. As the two basic characteristics for the two-parameter distributions are equal, the shapes of distribution density functions are almost identical; see solid lines in Fig 6. However, the PDFs are not identical, since the values of kurtosis *C*_*K*_ = *μ*_4_ / *μ*_2_ vary and equal to 5.67 and 3.68 for GE and IG distributions, respectively. As you move away from *C*_*V*_ = 0.41, the differences in the values of *C*_*S*_ of both distributions increase (Fig 4); therefore, the shapes of their density functions differ from each other. This is exemplified by *C*_*V*_ = 0.8 and corresponds to the values *C*_*S*_ = 1.69 and *C*_*S*_ = 2.4 for GE and IG distributions, respectively; see solid lines in Fig 6.

## Discrimination Procedures

The main disadvantage of using the wrong form of distribution for a flood series is that it over- or under-designs the hydraulic structures. Even if the sample size is not sufficiently large for making a proper choice among alternative distribution functions, a selection method is still needed and moreover all available information should be utilized for it. To find the best fitting model to empirical data from the set of competing models, a discrimination procedure is required. It must define a test statistics as well as a decision rule indicating the action to be taken for the sample under consideration. One can also prioritize all competing models according to the values of the selection criterion. However, the use of a discrimination procedure without the knowledge of its performance for the considered set of PDFs may be “a foolhardy gamble” [40] and may lead to erroneous conclusions [21]. To increase the efficiency of the model selection techniques in FFA, the use of several discrimination procedures along with the knowledge of their efficiency for a particular case is advisable.

### *K* procedure

The *K* procedure [41], [42] of model selection is based on the likelihood functions $L_{i}=\overset{N}{\underset{j=1}{\Pi }}f_{i}\left(x_{j}\right)$ for *i* = 1,…,*k* and *k* is the number of considered distributions expressed by their density functions *f*_*i*_. In fact, the *K* procedure is equivalent to the Akaike information criterion (AIC) [43] for distributions with the same number of parameters. The procedure points out the model with the highest value of the logarithm of the likelihood function as the true or the closest to the true model among all competing models, i.e.:

$$\underset{i=1,\ldots ,k}{\text{max}}\left[\underset{\hat{\theta }}{\text{max}}\left\{\text{ln}\;L_{i}\left(\hat{\theta }\right)\right\}\right]$$ (6)

where $\hat{\theta }$ is a set of distribution parameters evaluated by any estimation method. In this study, three methods of the assessment of parameters and, thus, of flood quantiles are applied, i.e. the method of moments (MOM) (e.g. [44]), the method of linear moments (LMM) (e.g. [19]), and the maximum likelihood method (MLM) (e.g. [45]). These methods were applied for the IG and GE distributions in [27] and [46], respectively. The accuracy of the estimates of large quantiles obtained from these three methods for the two- and three-parameter log-normal and GEV distributions have been analyzed in [47] both in the case of true and false hypothetical models, while the asymptotic bias of a quantile caused by the wrong distributional assumption has been analytically derived for a wide set of two-parameter distributions in [48], [49] and [17].

### *QK* procedure

The *QK* discrimination procedure bases on the statistics that is invariant under scale transformation of the data [30]:

$$S_{i}=\int_{0}^{\infty }f_{i}\left(\lambda x_{1},\ldots ,\lambda x_{N}\right)\lambda^{N-1}d\lambda$$ (7)

where *N* is the sample size and *f*_*i*_ is the probability density function with scale parameter equal to one for *k* alternative models, *i* = 1,…,*k*. The unknown shape parameter of each of the considered distributions is estimated by the MLM method and substituted into Eq 7. As the selection rule, Quesenberry and Kent [50] proposed to choose the model which corresponds to the highest value of the *S*_*i*_ statistics among competing PDFs. They showed that the *QK* discrimination procedure minimizes the sum of the probabilities of selecting the incorrect families of distribution. In practice the logarithm of the selection statistics *S*_*i*_ instead of the statistics itself is usually applied:

$$\underset{i=1,\ldots ,k}{\text{max}}\left[\text{ln}\;S_{i}\right]$$ (8)

The analytical formula for the logarithms of the *S*_*i*_ statistics of the inverse Gaussian distribution has been derived and published with small editorial error in [21]. Therefore, its corrected form is given below:

$$\begin{array}{l} \text{ln}\;S_{IG}=\text{ln}\left(2\right)+\frac{N}{2}\text{ln}\left(\frac{\beta }{\pi }\right)+2N\beta -\frac{3}{2}N\;\bar{\text{ln}\left(x\right)}+\frac{N}{4}\left(\text{ln}\left(\bar{x}\right)-\text{ln}\left(\bar{x^{-1}}\right)\right)+\\ \;+\text{ln}\left\{K_{\frac{N}{2}}\left[2N\beta \sqrt{\bar{x}\cdot \bar{x^{-1}}}\right]\right\} \end{array}$$ (9)

where *K*_*ν*_ is the modified Bessel function of the second kind (e.g. [37]). Since we failed to get the analytical *QK* formula for the generalized exponential distribution, the selection statistics ln *S*_*GE*_ has been calculated numerically from the definition Eq 7 using the trapezoidal rule for approximation of the definite integral (e.g. [39]).

### *KS* procedure

The *KS* procedure employs the Kolmogorov-Smirnov statistics $D_{i}^{\text{max}}$ proposed by Kolmogorov [51]. The statistics is oriented to measure the goodness of fit between the hypothetical and empirical distributions and, in terms of probability of exceedance, it has the form (e.g. [52]):

$$D_{i}^{\text{max}}=\underset{j=1,\ldots ,N}{\text{max}}|p_{i}\left(x_{j:N}\right)-{\hat{p}}_{j:N}|$$ (10)

where *p*_*i*_(*x*_*j*:*N*_) expresses the theoretical probability of the *j*-th element of the non-ascending ordered random sample *x*_1:*N*_ ≥…≥ *x*_*N*:*N*_ from the *i*-th distribution (in the set of *k* alternative distributions) and ${\hat{p}}_{j:N}$ is its empirical probability given here by the Weibull formula:

$${\hat{p}}_{j:N}=j/\left(N+1\right)$$ (11)

The model selected is the one which corresponds to the lowest value of $D_{i}^{\text{max}}$ function among all considered models, i.e.:

$$\underset{i=1,\ldots ,k}{\text{min}}\left[D_{i}^{\text{max}}\right]$$ (12)

Statistics *D* ^max^ is typically used as the test statistics in the Kolmogorov-Smirnov test of goodness of fit a distribution to the data. An attractive feature of *D* ^max^ is that its distribution does not depend on the underlying CDF being tested [53].

### *R* procedure

Since no simple statistical model can reproduce the dataset in its entire range of variability, it seems to be a right idea that the shape of the distribution tail should be a leading statistics when choosing a hypothetical distribution. Some guidelines and procedures for selecting the class of distributions that provides the best fit to the sample extremes are presented, for example, in [54–55]. However, there is a problem with a small number of data from the scope of the tail.

The *R* procedure follows the thought of fitting the model to the data in the range of the distribution tail. The parametric methods of the estimation of a model density function are asymptotically unbiased, which means that the assessment of any model parameter tends to its exact value for the sample withdrawn from the population of known distribution function. Then, in particular, the estimate of any quantile converges to its true value. Basing on this rule, the differences between the estimates obtained from various methods have been used to assess model fitting to the sample. The procedure of model discrimination has been explicitly proposed in [22] and based on the difference between 1% quantile assessment $\left({\hat{x}}_{1\%}\right)$ provided by the method of moments and the maximum likelihood method. The 1% quantile assessment is the most commonly used design value and corresponds to a probability of exceedance *p* = 0.01 (i.e. *F* = 0.99), expressed as a percentage. According to the relation between the probability and return period (Eqs 2 and 3), the ${\hat{x}}_{1\%}$ determines the probable maximum flow which appears, on average, once in 100 years.

Here, two discrimination statistics, $R_{i}^{1}$ and $R_{i}^{2}$, are proposed, for *i* = 1,…,*k* and *k* being the number of competing PDFs:

$$R_{i}^{1}=\left|{\hat{x}}_{1\%\left(i\right)}^{MLM}-{\hat{x}}_{1\%\left(i\right)}^{MOM}\right|$$ (13)

$$R_{i}^{2}=\left|{\hat{x}}_{1\%\left(i\right)}^{MLM}-{\hat{x}}_{1\%\left(i\right)}^{LMM}\right|$$ (14)

where ${\hat{x}}_{1\%\left(i\right)}^{MOM},\;{\hat{x}}_{1\%\left(i\right)}^{LMM},\;{\hat{x}}_{1\%\left(i\right)}^{MLM}$ are the 1% quantiles estimated by the moment method, the linear moments method and the maximum likelihood method, respectively. For both discrimination statistics (Eqs 13 and 14), the best model is the one with their lowest value:

$$\underset{i=1,\ldots ,k}{\text{min}}\left[R_{i}^{1}\right]$$ (15)

$$\underset{i=1,\ldots ,k}{\text{min}}\left[R_{i}^{2}\right]$$ (16)

Note that for normal distribution the MOM and MLM methods are equivalent. Therefore, if the normal distribution is among the alternative distributions, it would be chosen by the *R* procedure. What's more, all three estimation methods also give the same estimate of the mean for gamma and the two-parameter inverse Gaussian distributions. This gives for these distributions the similarity of the estimates of quantiles for these three methods in the range of the main probability mass and, to a certain extent, for higher quantiles as well. Therefore, to use the *R* procedure properly, the knowledge of its performance for the considered set of PDFs is required.

## Evaluation of Efficiency of Discrimination Procedures

Each discrimination procedure is considered to be of universal use, i.e. can be applied for model selection among any set of alternative PDFs, regardless of the sample size. However, the real drawback appears when for a small or medium sample size the discrimination procedures tend to favour some alternative distributions.

**Fig 7:**
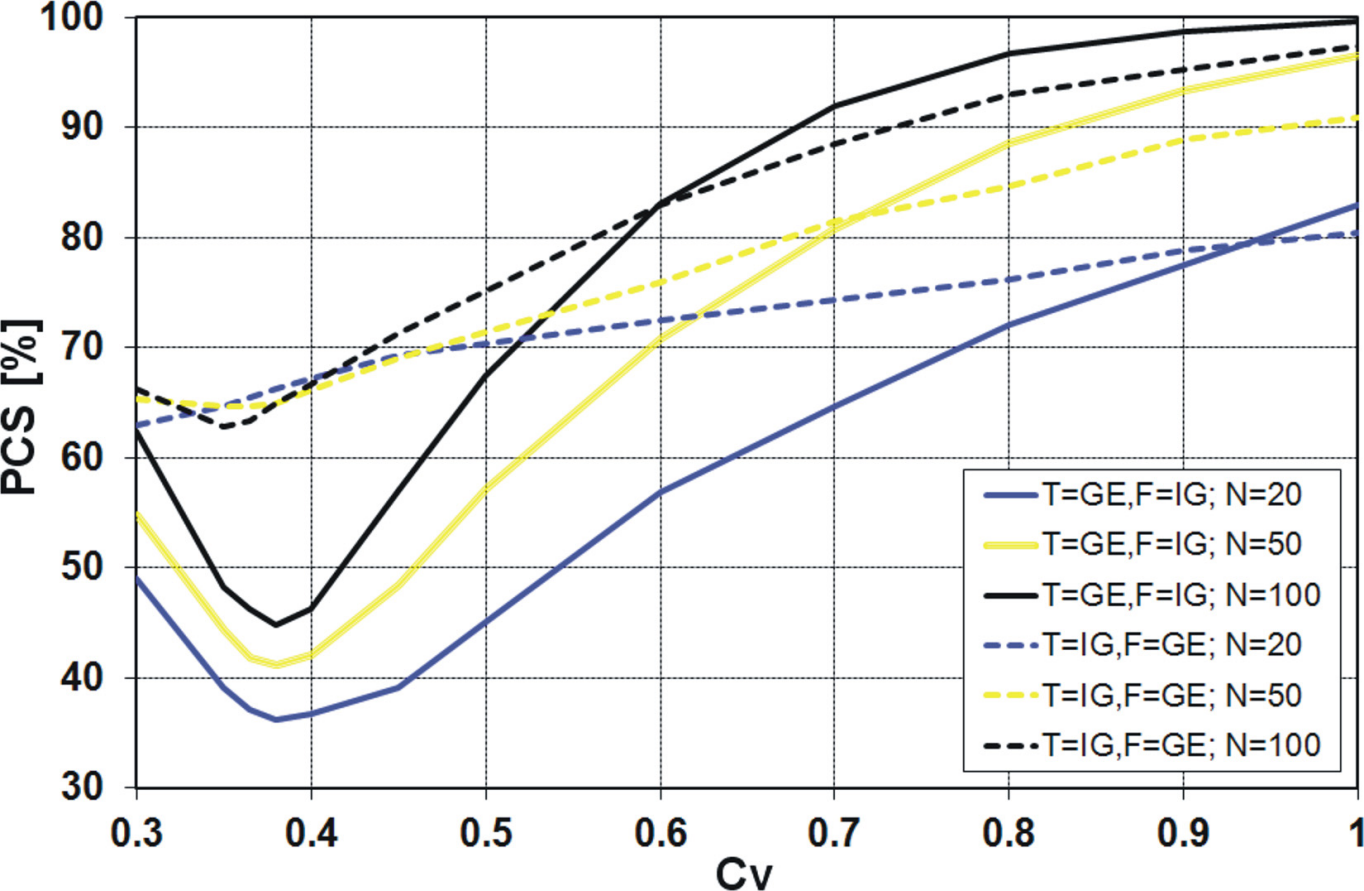
Probability of correct selection [%] for competing GE and IG distributions by the *K* discrimination procedures.

**Fig 8:**
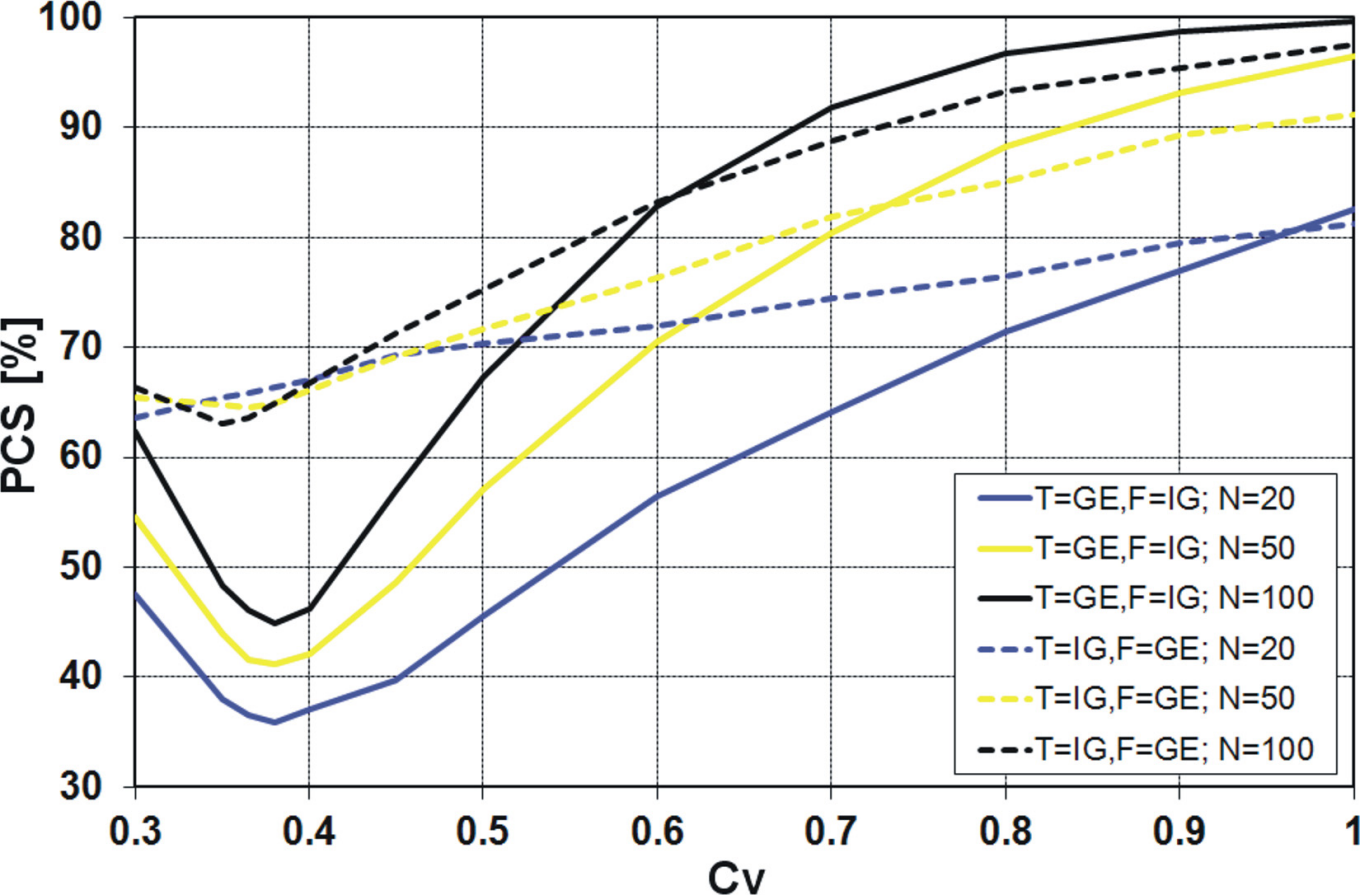
Probability of correct selection [%] for competing GE and IG distributions by the *QK* discrimination procedures.

**Fig 9:**
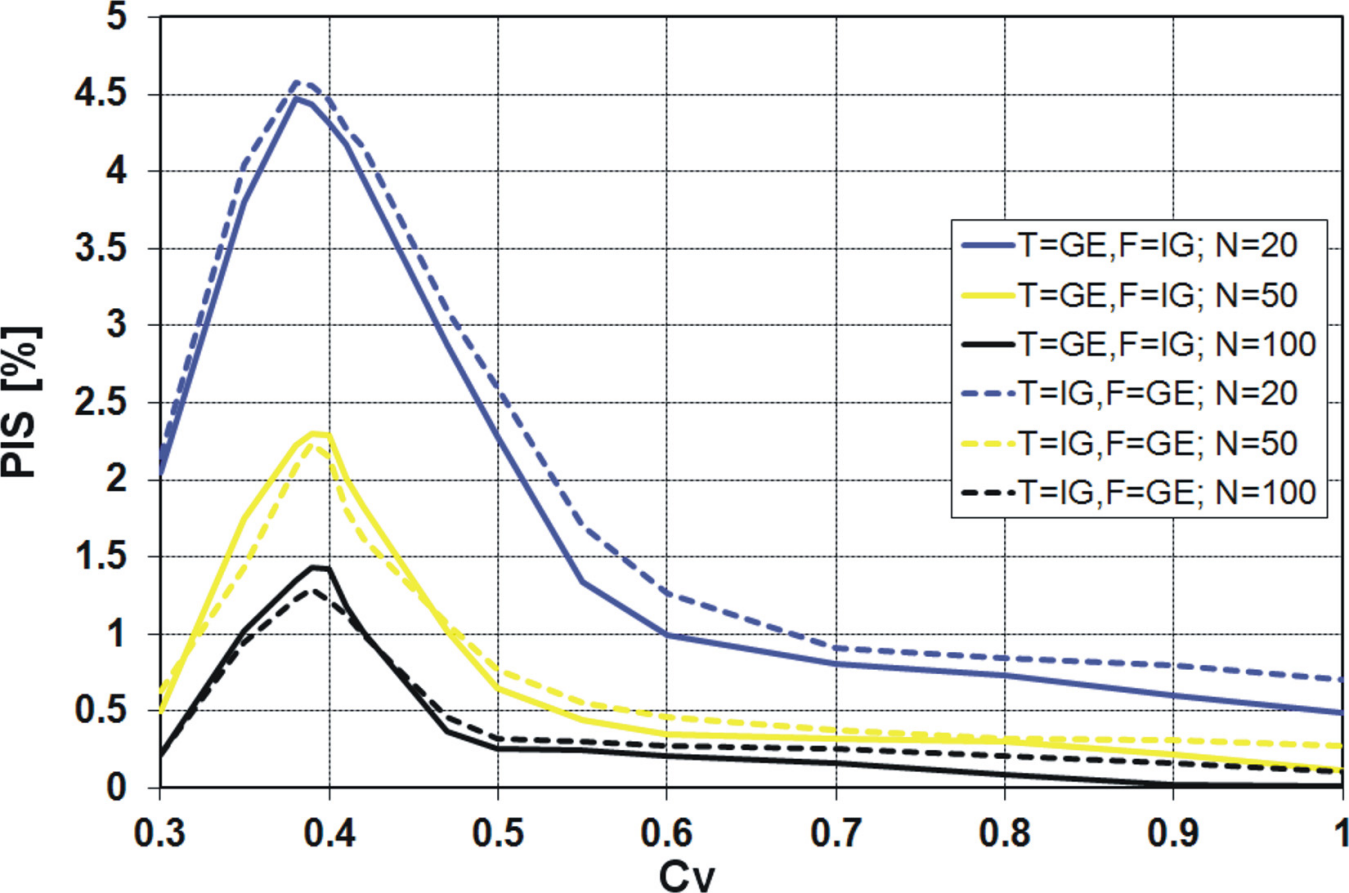
Probability of inconsistent selection [%] for competing GE and IG distributions by the *K* or *QK* discrimination procedures.

Discrimination between the generalized exponential and other two-parameter distributions has been already investigated in respect to the gamma [34], Weilbull [32] and log-normal [35] distributions. The ratio of the maximized likelihood functions has been used there to determine the probability of correct selection. Additionally, the selection among the We, LN and GE distributions has been studied in [56]. Here, the discrimination between the generalized exponential and the inverse Gaussian and vice versa is the subject of investigation. The efficiency of four procedures of discrimination has been evaluated using simulated data with GE as true (T) model and IG as a false (F) one and vice versa. *S* = 10,000 pseudo-random samples have been generated from the GE and IG distributions, respectively, for sample sizes *N* = 20, 50 and 100. To unify the distributions with respect to parameters, the original parameters were replaced by the mean *μ* and the variation coefficient *C*_*V*_. Without a loss of generality, the mean equal to one is assumed. The variation coefficient varies from 0.3 to 1.0; this covers the range of *C*_*V*_ values for Polish data (see Fig 1). The results available in the literature [21], [22] indicate significant differences in the values of the PCS obtained by the *K* and *QK* discrimination procedures for different pairs of distributions and small sample sizes generated. A similar result was expected for the pair of distributions IG and GE. However, to our surprise, the values of PCS according to the *K* discrimination procedure (Fig 7) are almost identical to the values from the *QK* procedure (Fig 8). The differences between these two procedures are shown in Fig 9.

The values of the probability of inconsistent selection **(**PIS) in Fig 9 mean that for a single sample generated from the assumed PDF, one of the procedures, *K* or *QK*, points out the right PDF (correct selection), while, at the same time, the latter procedure points out the wrong PDF (incorrect selection). In other words, the values of PIS are the percentages of inconsistency of the two procedures.

We have detected the identity of the *K* and *QK* procedures for the pairs of the inverse Gaussian with log-normal or gamma distributions. This issue will be further investigated. However, it seems that the *K* and *QK* procedures of discrimination are equivalent when IG is one of the alternative distributions, which is a unique feature of this distribution.

**Fig 10:**
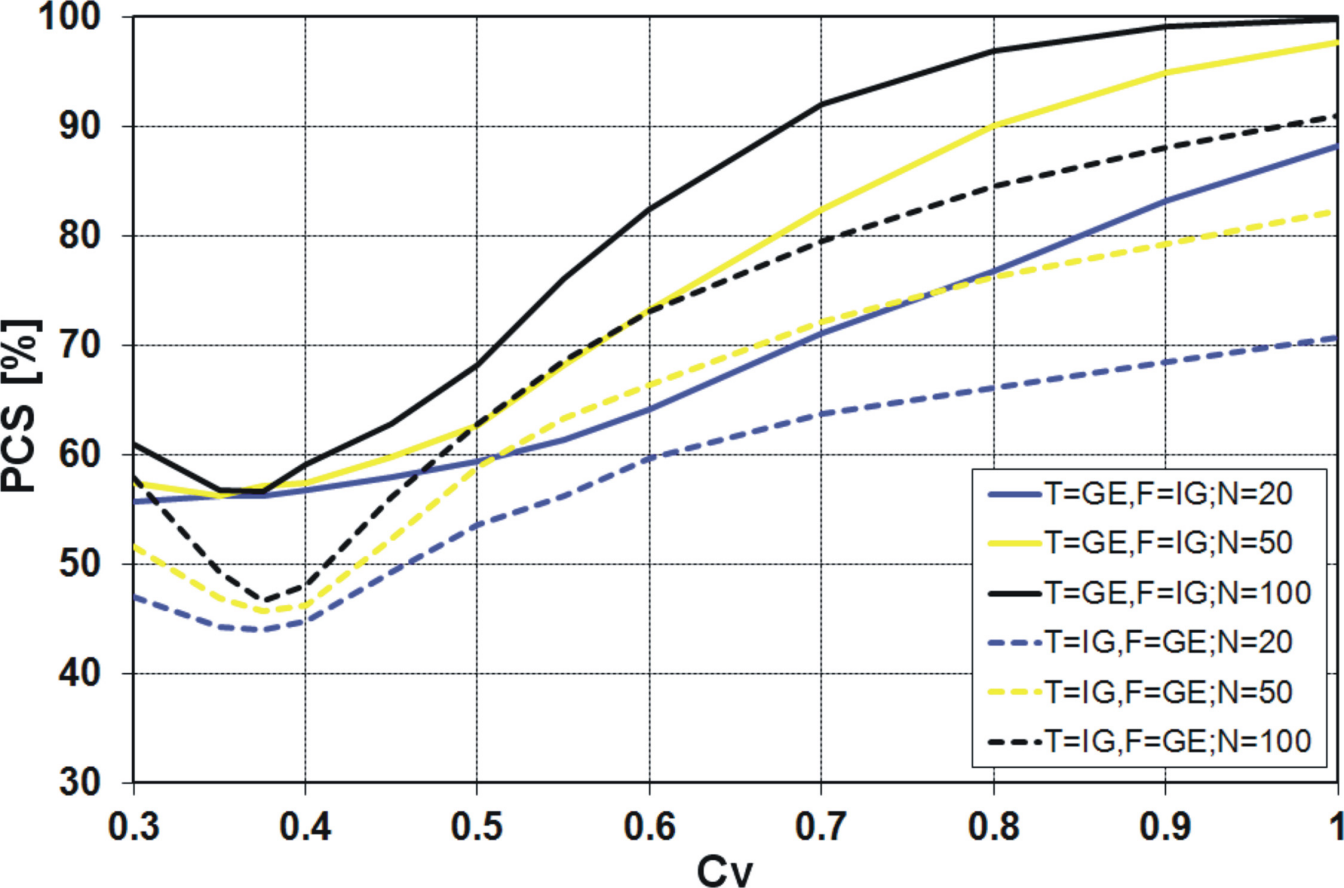
Probability of correct selection [%] for competing GE and IG distributions by the *KS* discrimination procedure.

**Fig 11:**
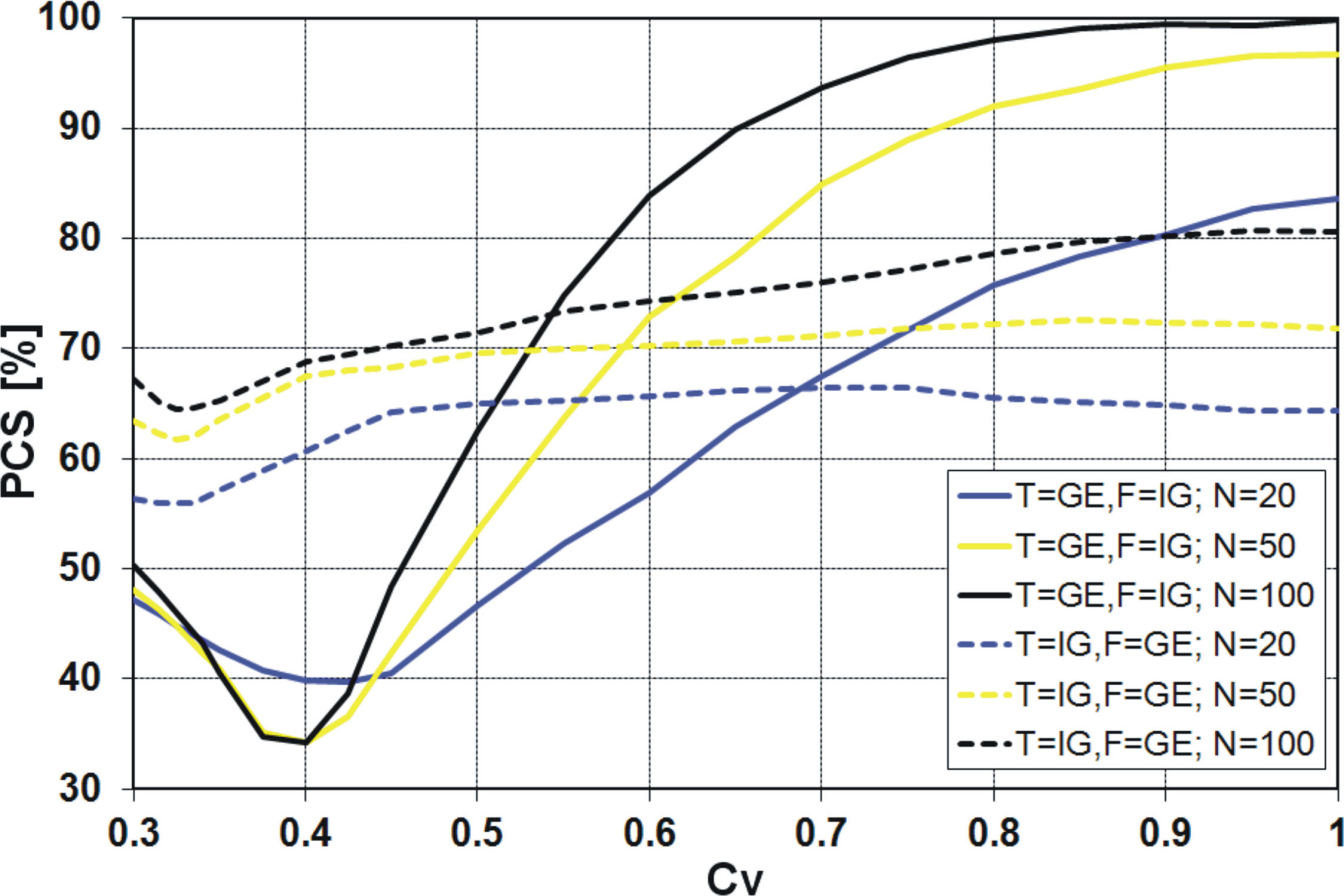
Probability of correct selection [%] for competing GE and IG distributions by the *R* ^1^ discrimination procedures.

**Fig 12:**
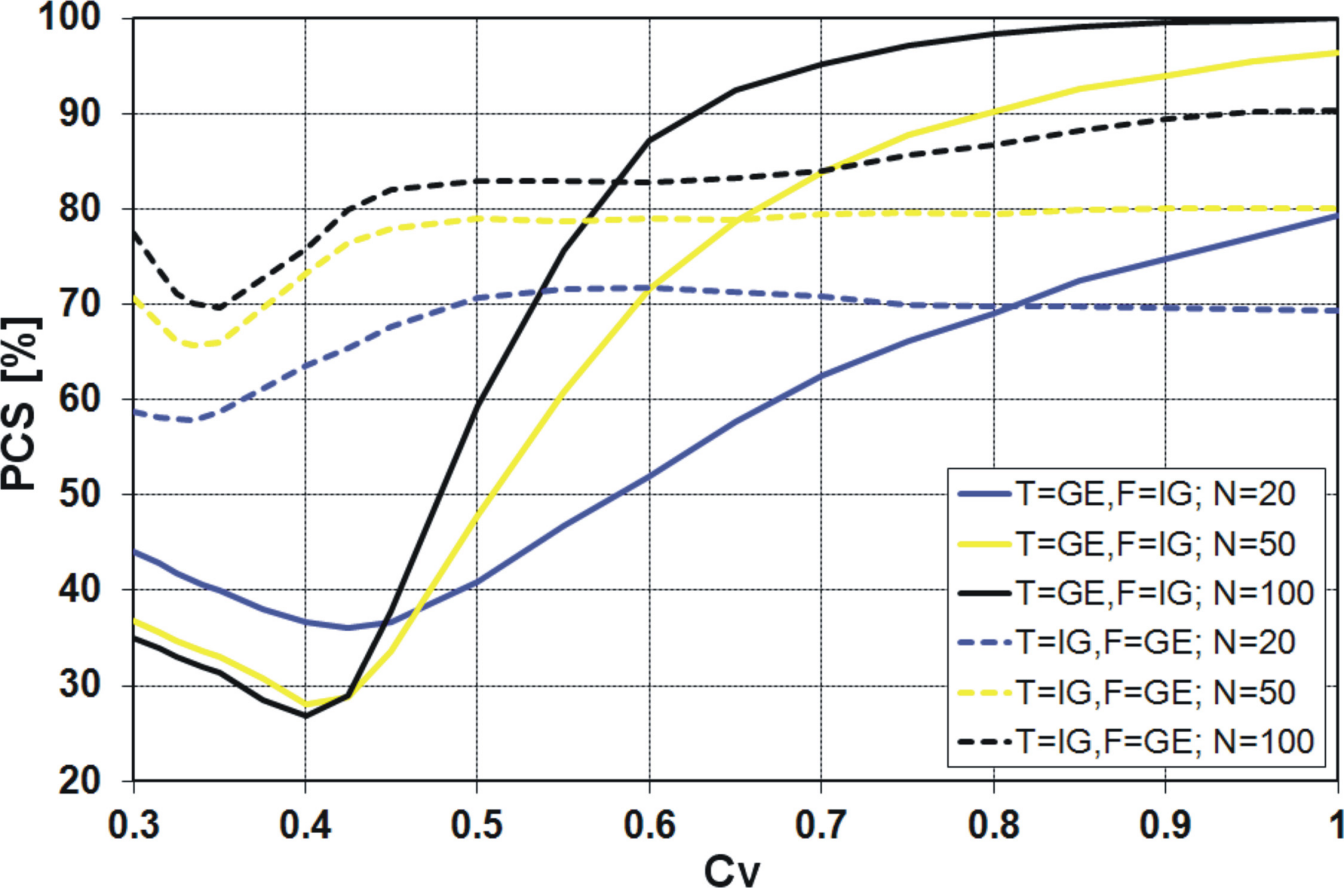
Probability of correct selection [%] for competing GE and IG distributions by the *R* ^2^ discrimination procedures.

Finally, the results obtained from the *KS* and both variants of *R* discrimination procedures, i.e. *R* ^1^ and *R* ^2^, are presented in Figs 10–12, respectively.

### PCS for the pair of GE and IG distributions

It is quite clear from the above figures that the PCS increases with increasing sample size, i.e. the probability of correct selection is the smallest for 20-element samples and the highest for 100-element samples. The exception is the *R* ^2^ procedure applied for the true GE distribution with IG as an alternative within the range of variation coefficient *C*_*V*_ from 0.3 to 0.45 (Fig 12). It is also clear that as *C*_*V*_ moves away from the value around 0.4, the PCS increases. For all considered discrimination procedures, if the variation coefficient is about 0.4, a sharp decline of the PCS value is visible. Only for the *KS* procedure the decrease of PCS value is more evident when IG is the true sample distribution (Fig 10), while for the other procedures this effect is stronger when GE is the true sample distribution (Figs 7, 8, 11 and 12). For example, for the *K* and *QK* procedures, when the data are drawn from the IG distribution, the PCS is about 63% at the minimum point for all the considered sizes of the sample, while if the data are drawn from the GE distribution, the PCS decreases up to about 34% for the sample size *N* = 20, i.e. then 66% of samples generated from GE distribution will be wrongly recognized as originated from IG parent distribution. The lowest value of the PCS among the GE and IG models for *C*_*V*_ ∼ 0.4 is related with the fact that for the variation coefficient equal to 0.41, the skewness coefficients *C*_*S*_ of both distributions are the same and amount to 1.23. Hence, for the range of *C*_*V*_ around 0.4, the investigated distributions have a similar shape, as shown in Fig 6.

Similar results as for the procedures *K* and *QK* are obtained for the procedure *R* (Figs 11 and 12). However, note that for the variant *R* ^2^ (Fig 12), the minimum of the PCS obtained for the case of T = GE and F = IG decreases below 30%. In general, if GE is the true sample distribution and *C*_*V*_ is lower than 0.5, it does not make sense to use any variant of *R* discrimination procedure, since the probability of the correct selection of the distribution is lower than 50%. Then the decision based on “head and tail” rule is more efficient and easier to use. The same applies to the procedures *K* and *QK* and *C*_*V*_ about 0.4, depending on the sample size *N*.

For all four discrimination procedures, the generalized exponential model is better recognizable than the inverse Gaussian, for moderate and large *C*_*V*_ values, i.e. *C*_*V*_ > 0.5, while for small *C*_*V*_ values, i.e. *C*_*V*_ < 0.5, the GE model is favoured only by *KS* procedure (Fig 10) and IG model is favoured by *K*, *QK* and *R* procedures (Figs 7, 8, 11 and 12).

For the range of *C*_*V*_ from 0.6 to 0.8, which covers most data of Polish Rivers, the probability of correct selection among GE and IG distributions is quite large. Except of some cases of 20-element series drawn both from the IG and GE distributions, the PCS is higher than 70%. However, it should be remembered that the above experiment relates to a special theoretical case when one of the two competing distributions is the true one. If there are more alternative models, the probability of the selection of the true distribution significantly decreases. Similarly, the PCS is smaller if a set of alternative distributions consists of the PDFs which are similar in type to the true distribution. Note, the exponential distribution is a special case of the gamma, Weibull and generalized exponential models, if the shape parameter is equal to one, being a special case of Pareto, if the shape parameter is equal to zero. Then the PCS among any pair of the distributions from the set above would be lower than the PCS among GE and IG distributions.

## Asymptotic Model Error in Respect to the Upper Quantile

In flood frequency analysis, the assumed (hypothetical) model is treated as the correct (true) model and any assessment of the accuracy of the estimation of its parameters and quantiles is usually made assuming that the considered random sample is derived from that probability distribution. In this way, the error of the choice of false distribution, i.e. the model error, is omitted, although this error can have a significant impact on the accuracy of the quantiles estimation. For a given estimation method, the total bias of quantile estimate consists of a sampling bias which asymptotically converges to zero and a model bias caused by wrong distributional selection. Those biases can be of opposite signs. The theoretical background for the asymptotic bias caused by false distributional assumption for various estimation methods has been presented in [48] followed by derivations for various pairs of (True, False) distributions in [49], [17].

Here, the set of competing PDFs involves the generalized exponential and inverse Gaussian distributions and the interest is in the derivation of the asymptotic model bias of the 1% quantile estimate; when the GE is the true (T) population model, then the IG is falsely (F) adopted for the hypothetical PDF, and vice versa. Three estimation methods presented in section 3.1 are used as approximation method of T distribution by F distribution. The relative model bias is defined for each approximation method as:

$$RB\left({\hat{x}}_{1\%}\right)=\frac{{\hat{x}}_{1\%}\left(F/T\right)-{\hat{x}}_{1\%}\left(T\right)}{{\hat{x}}_{1\%}T}$$ (17)

**Fig 13:**
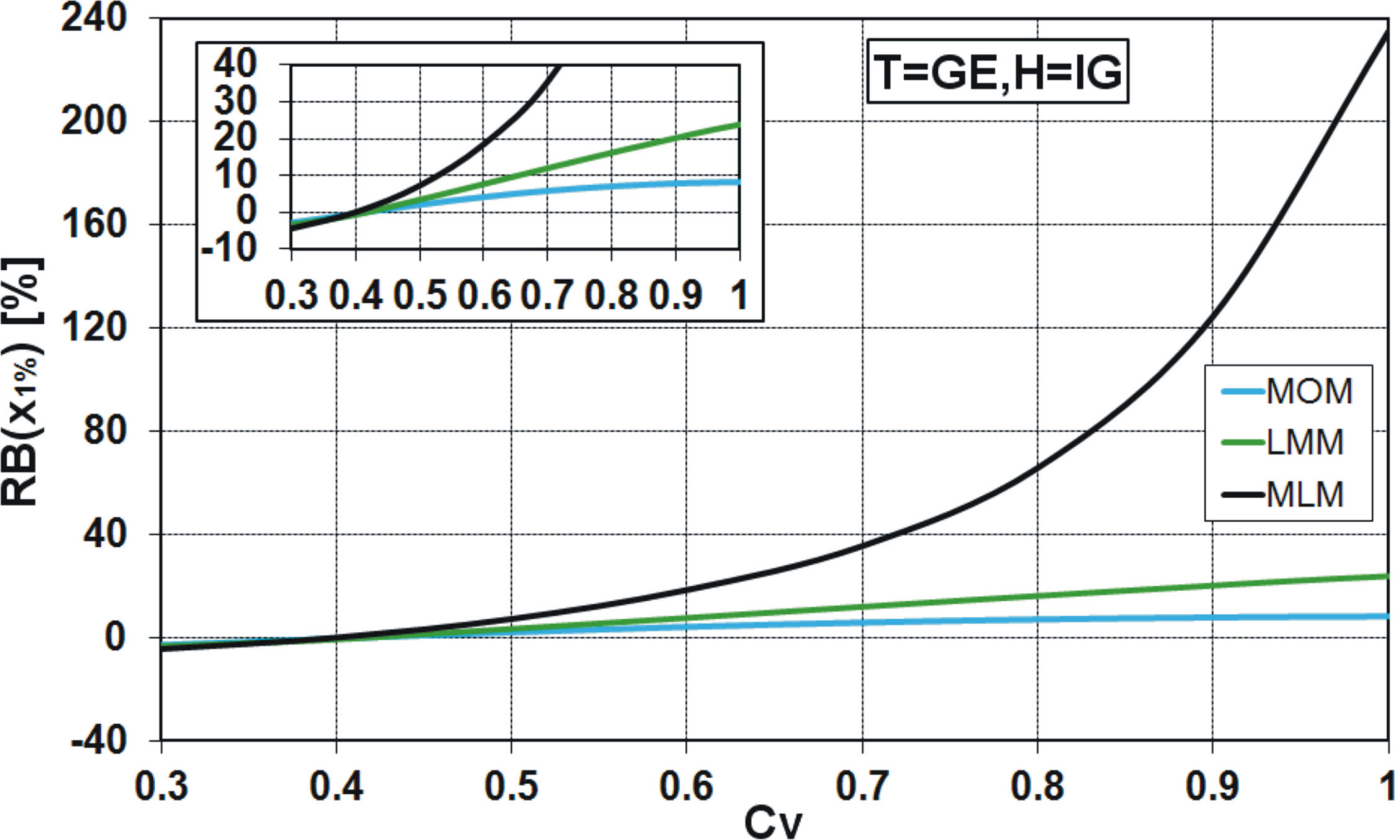
Relative asymptotic bias [%] of ${\hat{x}}_{1\%}$ from T = GE distribution, assuming F = IG model.

**Fig 14:**
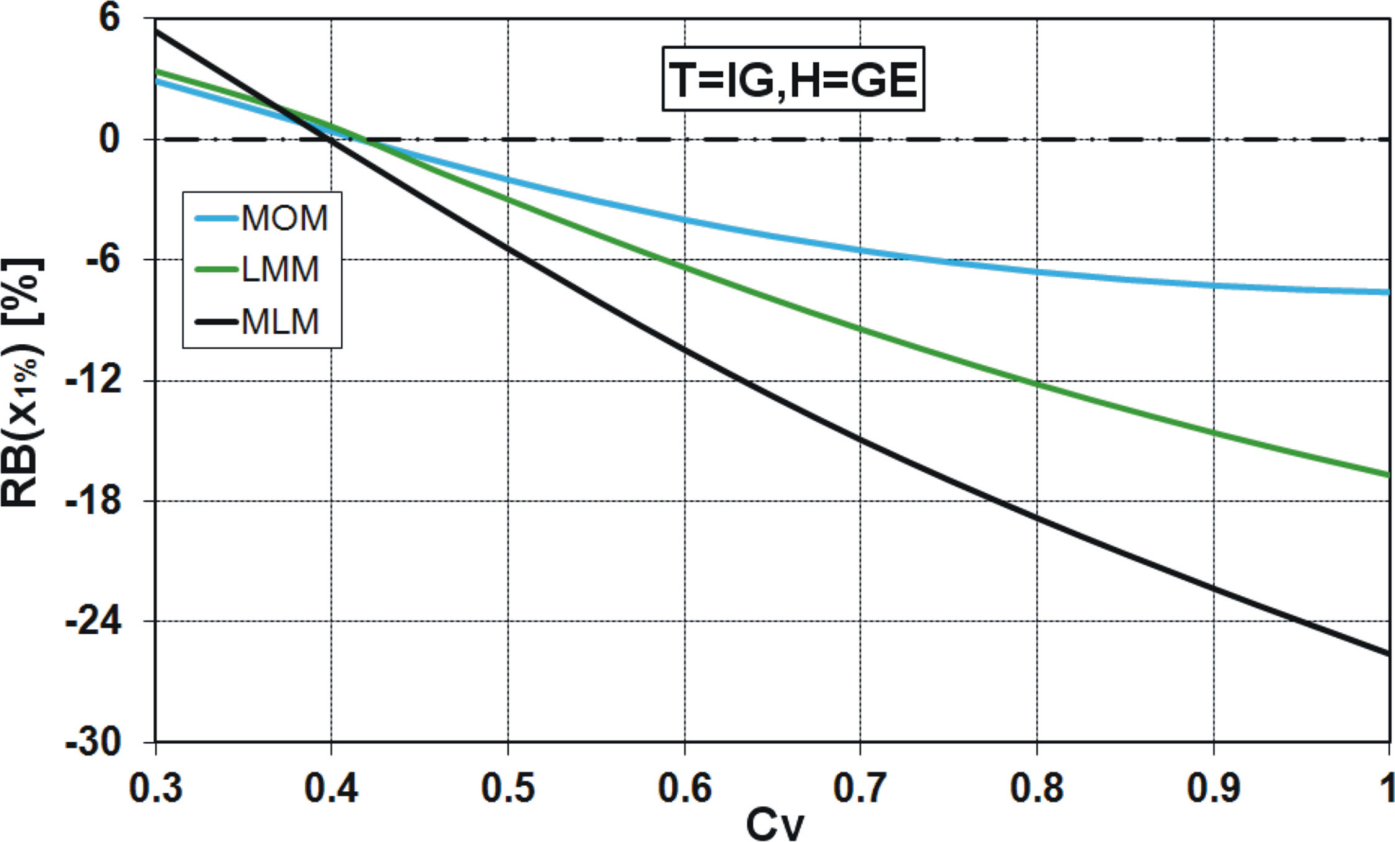
Relative asymptotic bias [%] of ${\hat{x}}_{1\%}$ from T = IG distribution, assuming F = GE model.

To present a unified treatment for both distributions and three estimation methods, the mean of the T distribution equal to 1.0 and the variation coefficient *C*_*V*_ varying from 0.3 to 1.0 are considered in the experiment. The relative asymptotic bias of ${\hat{x}}_{1\%}$ is determined analytically by the methods of MOM and LMM, while to use MLM, the samples of size *N* = 9000 have been generated from the GE and IG distributions, in turn. The results are presented in Figs 13 and 14.

One can see that the incorrect choice of distribution for describing the chosen data series may lead to large errors of the 1% quantile estimate, especially if the maximum likelihood method is applied. For example, if the large sample from the GE distribution of the variation coefficient *C*_*V*_ = 0.8 is falsely modelled by the IG distribution, then the relative asymptotic bias of ${\hat{x}}_{1\%}$ equals 7% and 16.2% for the MOM and LMM estimation methods, respectively, while it is nearly 66% for the MLM (Fig 13). In the opposite case, i.e. when the sample is derived from the IG distribution and the GE model is mistakenly assumed, the rank of estimation methods is similar, except that the bias sign is negative. For *C*_*V*_ = 0.8 and MLM estimation method, the $RB\left({\hat{x}}_{1\%}\right)$ is equal to -18.8%, while for LMM and MOM methods, the $RB\left({\hat{x}}_{1\%}\right)$ is equal to -12.2% and -6.59%, respectively (Fig 14). The differences in the value of $RB\left({\hat{x}}_{1\%}\right)$ obtained from the MLM and two other estimation methods are significant, especially in the case of T = GE, F = IG (Fig 13), being lower in the case of T = IG, F = GE (Fig 14). This finding essentially diminishes the practical usefulness of MLM in hydrological extremes analysis, because its efficiency may not compensate for the (frequently) large bias produced by the model misspecification, which is highly probable in hydrological reality.

## Case Study

In order to analyze the GE and IG distributions fitting to the Polish data, four gauging stations have been selected as examples. These are Rudze, Stróża, Koszyce Wielkie and Wadowice sections on the Wieprzówka, Raba, Biała and Skawa Rivers, respectively. Their basic characteristics are presented in Table 2 under the numbers 15, 16, 26 and 14, in turn. All four stations are located in the mountain area in the south part of Poland (Fig 3) and are characterized by a high dynamics of flows. For each gauge, the 90-year series of annual maximum flows from the period 1921–2010 has been investigated. Both two-parameter models, namely GE and IG, have been used to reproduce the data series. The 1% quantile has been estimated by three estimation methods, MOM, LMM and MLM. To find the best fitted distribution among the two competing PDFs, four discrimination procedures have been applied, namely *K*, *QK*, *KS* and *R*. Since the alternative distributions contain the same number of parameters, any procedure of model selection can be used for the assessment of the best fitting model. However, for a group of PDFs containing both two- and three-parameter functions, a discrimination procedure which takes into account the number of model parameters should be used, such as, for example, the Akaike information criterion (e.g. [43], [57]). Otherwise, the three-parameter distribution would always be better than their counterpart two-parameter models.

### Accuracy of the fit of models

The values of 1% quantile estimates for selected gauging stations are presented in Table 4.

**Table 4:** The 1% quantile estimates for selected gauging stations in Poland, assuming GE and IG distributions, respectively.

Gauging station	Estimation method	${\hat{x}}_{1\%}$ (GE)	${\hat{x}}_{1\%}$ (IG)
Rudze	MOM	200.08	212.81
	LMM	213.56	247.71
	MLM	220.38	330.09
Stróża	MOM	787.69	838.02
	LMM	795.59	908.90
	MLM	756.70	970.84
Koszyce	MOM	1000.7	1065.0
	LMM	1034.3	1193.5
	MLM	973.80	1248.1
Wadowice	MOM	956.85	1017.1
	LMM	958.83	1091.4
	MLM	894.97	1108.3

For each section, the values of ${\hat{x}}_{1\%}$ differ significantly between the distributions, when the MLM method is used for their estimation, while the differences are the smallest when the MOM method is applied. Generally, while estimating the upper quantiles, the MLM is the most sensitive in respect to model choice and the MOM is the most stable estimation method. To find which of the distributions is the best fitting for each annual maximum flow series and, in particular, to find which of the estimates of 1% quantile is the most reliable, the four procedures of discrimination are applied and their results are shown in Table 5. The bold font on a gray background means that for particular gauging stations the model is the best fitted among two alternative PDFs.

**Table 5:** Distribution choice by the four discrimination procedures for annual maximum records of selected gauging stations.

Discrimination procedure	Estimation method	Rudze	Rudze	Stróża	Stróża	Koszyce	Koszyce	Wadowice	Wadowice
		GE	IG	GE	IG	GE	IG	GE	IG
*K* procedure	MOM	**-451.54**	-482.78	**-565.82**	-568.16	**-587.89**	-589.08	-582.13	**-580.29**
	LMM	**-450.00**	-466.66	**-565.95**	-566.18	-588.30	**-586.47**	-582.17	**-579.51**
	MLM	**-449.86**	-457.33	**-565.60**	-565.73	-587.80	**-586.27**	-581.53	**-579.49**
*QK* procedure	MLM	**-451.39**	-458.78	**-567.27**	-567.36	-589.44	**-587.89**	-583.23	**-581.17**
*KS* procedure	MOM	**0.1052**	0.1500	**0.0670**	0.0733	**0.1097**	0.1219	**0.0656**	0.0850
	LMM	**0.0933**	0.1282	**0.0640**	0.0712	0.0958	**0.0776**	**0.0647**	0.0651
	MLM	**0.0918**	0.1656	0.0762	**0.0704**	0.1181	**0.0755**	0.0913	**0.0652**
*R* procedure	*R* ^*1*^	**20.298**	117.29	**30.989**	132.82	**26.929**	183.13	**61.871**	91.181
	*R* ^*2*^	**6.8190**	82.382	**38.889**	61.935	60.540	**54.553**	63.859	**16.917**

For the Rudze gauging station, all four procedures of discrimination point out the generalized exponential distributions as better fitted than the inverse Gaussian. The same is true for the Stróża station, with the exception of the *KS* discrimination procedure and the MLM estimation method when the IG distribution fits to the data series better. For the Koszyce and Wadowice sections, the choice of the best fitting model varies greatly, depending on the discrimination procedure and the estimation methods. In the case of the Koszyce station, in most variants of procedure and method (6 out of 9 cases), the IG distribution is pointed out as the better fitted than the GE. The superiority of the IG distribution over the GE is indicated only by the *K* and *KS* procedures along with the MOM method and by the variant *R* ^1^ of the *R* procedure. Similarly, the data series from the Wadowice gauging station should be modelled using IG distribution rather than GE, according to the *K*, *QK*, *KS* procedures with the MLM method and the variant *R* ^2^ of the *R* procedure. In the other three cases, the GE distribution over the IG distribution is favoured.

Despite the similar hydrological regime and the same observation period, for each gauging station, the best fitted model depends on the discrimination procedure and the estimation method. However, as shown above, the superiority of GE distribution over the previously dominant IG distribution, detected in many cases, proves that GE occupies one of the leading positions among distributions commonly used in flood frequency modelling of Polish data.

### Aggregation of models

The results of PCS studies and discrimination procedures confirm our belief as to the uncertainty of the identification of the true distribution type among alternative distributions. Besides, the general considerations lead to the conclusion that the true distribution type is beyond the cognitive capabilities. Even if the true distribution exists, it would probably have countless parameters, unidentifiable from the available observation series. In summary, we are inclined to believe that, for a set of alternative models, the quantile estimate obtained from each model contains a piece of information about the true quantile value. This piece of result should be provided with a proper weight, depending on the quality of the fit of a particular model to the data series. Such a multi-model approach (called “aggregation”) in the estimation of the extreme value distribution quantiles has been presented by Bogdanowicz [23]. The aggregated quantile $\left({\bar{x}}_{p\%}\right)$ is defined as a sum of quantiles estimated by MLM method for each of alternative distributions multiplied by their weights *w*_*i*_. The weights are defined using the likelihood function:

$$w_{i}=\frac{L_{i}}{\sum_{m=1}^{k}L_{m}}\;\text{for}\;i=1,\ldots ,k$$ (18)

Eq 18 is valid for the case when all distribution candidates have the same number of parameters; otherwise, the Akaike information criterion is applied for the definition of the distribution weights. The weights can be interpreted as the conditional probability of the adequacy of *i*-th model, so the aggregated quantile is a conditional expected value.

The aggregation data for four investigated gauging stations are presented in Table 6.

**Table 6:** The aggregation of 1% quantile of annual maximum flow series for selected gauging stations in Poland.

Gauging station	weight	weight	${\hat{x}}_{1\%}$ (GE)	${\hat{x}}_{1\%}$ (IG)	${\bar{x}}_{1\%}$
	GE	IG			
Rudze	0.9994	0.0006	220.38	330.09	220.44
Stróża	0.5325	0.4675	756.70	970.84	856.81
Koszyce	0.1784	0.8216	973.80	1248.1	1199.2
Wadowice	0.1150	0.8850	894.97	1108.3	1083.8

The estimates of upper quantiles obtained from the aggregation method seem to be more reliable and stable than from the classical approach, since the aggregation allows to partly overcome the problem of the arbitrary choice of the best fitted model. Moreover, the aggregation of models mitigates the problem of fluctuations of the upper quantile estimates, used as the hydrological design value along the river.

## Summary and Conclusions

Flood frequency analysis has been used for designing hydrological structures for over the century. Despite many distributions proposed for fitting the flood extremes data, the analysis of the annual maximum flow series for Polish Rivers reveals that the inverse Gaussian and generalized exponential distributions seem to be a desirable complement.

Applying a discrimination procedure without the knowledge of its performance for the considered PDFs may lead to erroneous conclusion and finally to erroneous quantile estimates. The experiment on the probability of correct selection (PCS) reveals that the values of PCS are fairly high for *K*, *QK*, *KS* and *R* procedures of discrimination. However, they sharply decrease in the vicinity of the variation coefficient 0.4. Applying the *K*, *QK* and *R* procedures, the PCS is even lower than 50% if the GE is the true sample distribution, being higher than 60% if the IG is the true sample distribution. If the probability of selection of the right PDF is mostly much lower than 50%, which is observed here for the range of *C*_*V*_ between 0.3 and 0.5, it does not make sense to employ the sophisticated procedures of discrimination, since a simple “head and tail” rule is more efficient and easier to use. However, for the range of *C*_*V*_ from 0.6 to 0.8, which covers most data of Polish Rivers, the probability of correct selection among GE and IG distributions is quite large for all four discrimination procedures, being higher than 70% for moderate and large sample sizes.

The analysis of fitting the generalized exponential and inverse Gaussian distributions to the 90-year series of annual maximum flows for four selected gauging stations in Poland, reveals that the assessment of 1% quantile differs considerably for various models and estimation methods. The choice of the best fitting model (distribution type and its parameter values) is not unique. It depends on the discrimination procedure used (criterion for the selection of the distribution) and the method of estimation. It is characteristic for hydrological size of samples. The results from four procedures of discrimination applied to modelling of annual peak flow series for four Polish gauging stations show in many cases the superiority of GE distribution over IG distribution, which has been dominant in FFA in Poland so far. This shows that GE occupies one of the leading positions among distributions commonly used in flood frequency modelling of Polish data and can be included into the group of the alternative distributions. The solution to the problem of the choice of the best fitting model can be the aggregation of quantiles obtained from all candidate distributions.

Despite the use of multiple distributions for flood frequency analysis, there is still a room for new models. However, one should remember that the choice of the distribution is just one aspect of the modelling of flood frequency, besides the choice of the estimation method and discrimination procedures. As shown in the paper, the selection of each of the above elements does have a significant impact on the estimate of desirable quantile. Moreover, note that the proliferation of statistical techniques causes the heterogeneity of results and finally leads to an increase the uncertainty of flood quantile estimates, instead of leading to clear solution. This stands in contrast with the expectation of engineers and hydrologists as they want to have a unique value, not accepting the uncertainty.

## Appendix

### Derivation of the third linear moment *λ*_3_ for GE distribution

The cumulative distribution function of the three-parameter generalized exponential distribution has the form:

$$F(x)={\left(1-e^{-\lambda \left(x-\varepsilon \right)}\right)}^{\alpha }$$ (A.1)

where *ε*, *λ*, *α* > 0 are the location, scale and shape parameters, respectively. Hence we get the following quantile:

$$x=\varepsilon -\frac{\text{ln}\left(1-F^{1/\alpha }\right)}{\lambda }$$ (A.2)

The third linear moment can be defined using the formula [19]:

$$\lambda_{3}=6\beta_{2}-6\beta_{1}+\beta_{0}$$ (A.3)

where *β*_*r*_, for *r* = 0,1,2,…, are the probability weighted moments of a random variable:

$$\beta_{r}=\int_{-\infty }^{+\infty }xF^{r}\left(x\right)\;dF\left(x\right)$$ (A.4)

Substituting Eqs A.1 and A.2 to Eq A.4 for *r* = 0,1,2, we get the probability weighted moments of forms:

$$\beta_{0}=\mu =\varepsilon +\frac{\psi \left(\alpha +1\right)-\psi \left(1\right)}{\lambda }$$ (A.5)

$$\beta_{1}=\frac{1}{2}\left(\varepsilon +\frac{\psi \left(2\alpha +1\right)-\psi \left(1\right)}{\lambda }\right)$$ (A.6)

$$\beta_{2}=\frac{1}{3}\left(\varepsilon +\frac{\psi \left(3\alpha +1\right)-\psi \left(1\right)}{\lambda }\right)$$ (A.7)

where *ψ* is the digamma function [37], [38].

Finally, after substituting Eqs A.5–A.7 into Eq A.3 and after some simplifications, the formula of the third linear moment for GE distribution has a form:

$$\lambda_{3}=\frac{\psi \left(\alpha +1\right)-3\cdot \psi \left(2\alpha +1\right)+2\cdot \psi \left(3\alpha +1\right)}{\lambda }$$ (A.8)

## Supporting Information

S1 Table Abbreviations and symbols commonly used in the paper. (DOC)
