# Supplementary material for: Generalized Exponential Distribution in Flood Frequency Analysis for Polish Rivers
Source: PLoS One. 2015 Dec 10;10(12):e0143965. doi: 10.1371/journal.pone.0143965 (PMC4684336; doi:10.1371/journal.pone.0143965)
Supplement: S1 Table — (DOC) [file pone.0143965.s001.doc]

S1 Table. Abbreviations and symbols commonly used in the paper.

| Abbreviation/symbol  o  or | Explication |
| --- | --- |
| FFA | Flood frequency analysis |
| **PDF** | **Probability density function** |
| CDF | Cumulative distribution function |
| GE | Generalized exponential distribution |
| IG | Inverse Gaussian distribution |
| Ga | Gamma distribution |
| We | Weibull distribution |
| LN | Log-normal distribution |
| LL | Log-logistic distribution |
| LG | Log-Gumbel distirbution |
| $\mu_{r}$ | Conventional moments, $r=1,2,3,...$ |
| $\lambda_{r}$ | Linear moments (*L*-moments), $r=1,2,3,...$ |
| $C_{V}$ | Variation coefficient |
| $LC_{V}$ | Linear Variation coefficient |
| $C_{S}$ | Skewness coefficient |
| $LC_{S}$ | Linear skewness coefficient |
| PCS | Probability of correct selection |
| $x_{F}$ | **Quantile of the order of** $F\in\left(0,1\right)$ |
| $\hat{x}_{1\%}$ | Estimated quantile of **a probability of** **exceedance** $p=0.01$ ($F=0.99$)  **(**$F=0.99$expressed **as a percentage**). |
| $\bar{x}_{p\%}$ | Aggregated quantile of **a probability of** **exceedance** $p\in\left(0,1\right)$ |
| MOM | **Method of moments (for estimation)** |
| LMM | **Method of linear moments (for estimation)** |
| MLM | **Maximum likelihood method (for estimation)** |
| *K* procedure | Discrimination procedure based on the likelihood functions $L$ |
| *QK* procedure | Discrimination procedure based on the statistics $S$ proposed  by Quesenberry and Kent |
| *KS* procedure | Discrimination procedure based on the Kolmogorov-Smirnov statistics $D^{\max}$ |
| *R* procedure | Discrimination procedure based on the statistics $R^{1}$ or $R^{2}$,  i.e. based on the difference between 1% quantile assessments |
| $R^{1}$ | Difference between 1% quantile assessment estimated by the moment method and the maximum likelihood method |
| $R^{2}$ | Difference between 1% quantile assessment estimated by the linear moments method and the maximum likelihood method |
| $RB\left(\hat{x}_{1\%}\right)$ | Relative asymptotic bias of $\hat{x}_{1\%}$ |
